# Supplementary material for: Epsin-mediated degradation of IP3R1 fuels atherosclerosis
Source: Nat Commun. 2020 Aug 7;11:3984. doi: 10.1038/s41467-020-17848-4 (PMC7414107; doi:10.1038/s41467-020-17848-4)
Supplement: Supplementary file 1 — Supplementary Information [file 41467_2020_17848_MOESM1_ESM.pdf]

Supplementary Figures

Epsin-mediated degradation of IP3R1 fuels atherosclerosis

Yunzhou Dong[1], Yang Lee[1], Kui Cui[1], Ming He[2], Beibei Wang[1], Sudarshan Bhattacharjee[1], Bo Zhu[1], Tadayuki Yago[3], Kun Zhang[1], Lin Deng[4], Kunfu Ouyang[2], Aiyun Wen[1], Douglas B. Cowan[1,5], Kai Song[1], Lili Yu[1] , Megan L. Brophy[1], Xiaolei Liu[6], Jill Wylie-Sears[1], Hao Wu[1], Scott Wong[1], Guanglin Cui[7], Yusuke Kawashima[8,9], Hiroyuki Matsumoto[8], Yoshio Kodera[9], Richard J.H. Wojcikiewicz[10], Sanjay Srivastava[11], Joyce Bischoff[1], Da-Zhi Wang[5], Klaus Ley[12], and Hong Chen[1*]

* To whom correspondence should be addressed: Hong Chen, Ph.D., Vascular Biology Program, Boston Children's Hospital, Harvard Medical School, Boston, MA 02115. Tel: 617-919-6304, Fax: 617-730-0232, hong.chen@childrens.harvard.edu

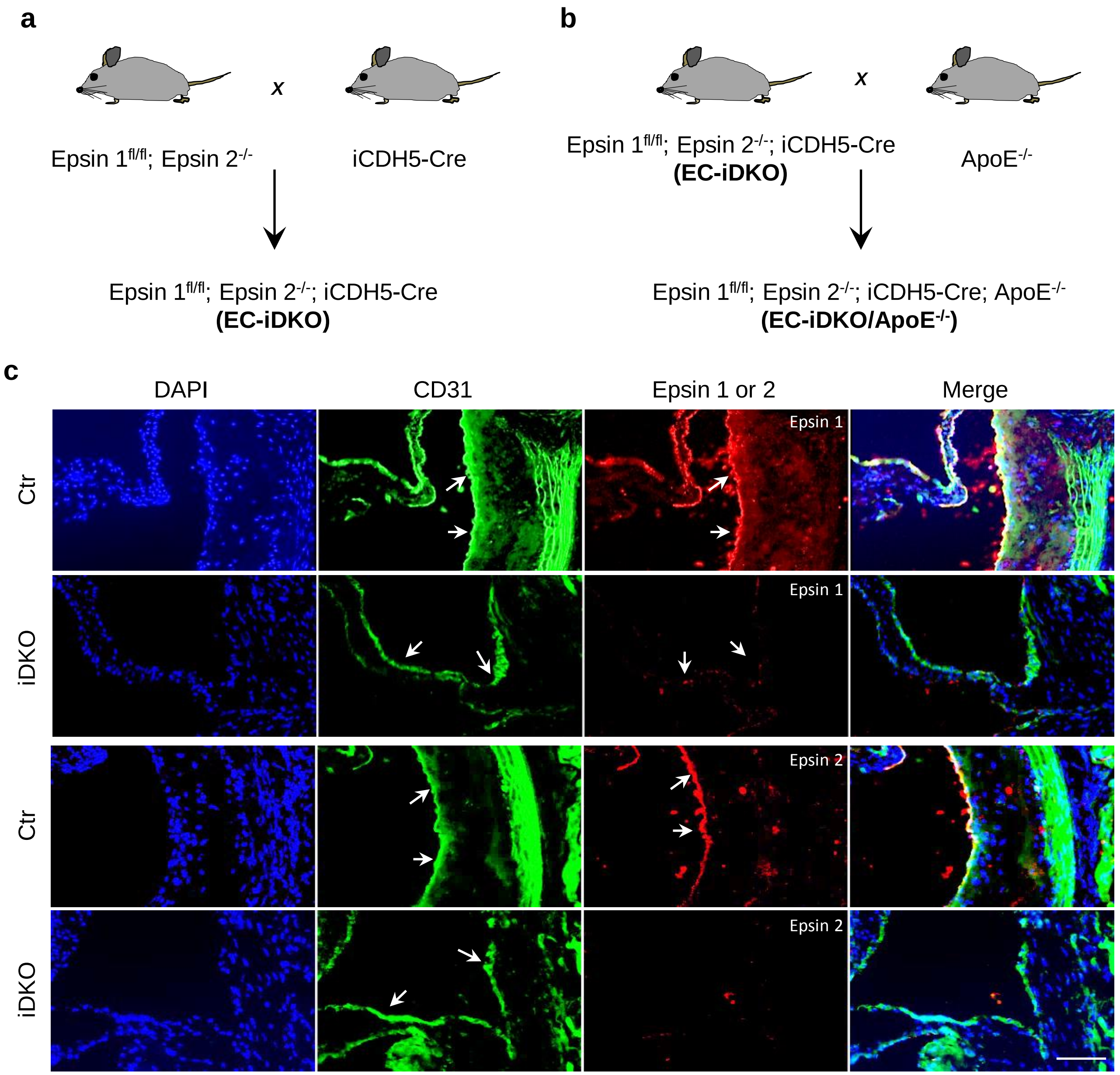

Supplementary Figure 1 Strategy for generating endothelial-specific, inducible double knock-out mice and confirmation of epsin deletion. **a, b** Various mice were crossed to establish the EC-iDKO/ApoE$^{-/-}$ (iDKO) strain. EC-iDKO mice were first established (**a**) and breed with ApoE$^{-/-}$ mice (**b**). **c** Characterization of the endothelium deletion of epsins using specific epsin 1 or epsin 2 antibodies by immunofluorescent staining in Control and EC-iDKO aortic roots. Scale bar: 100 µm.

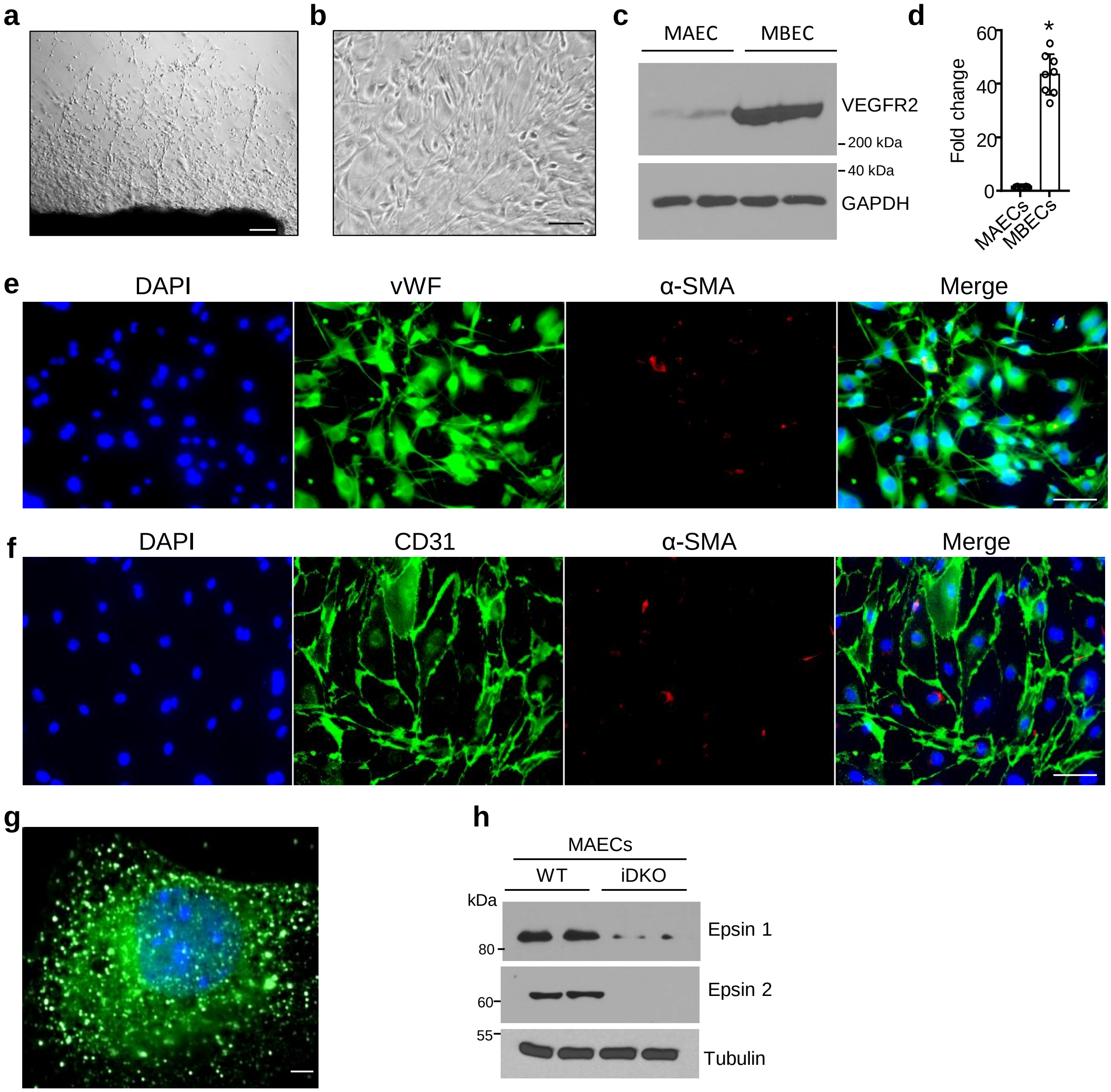

Supplementary Figure 2 Isolation and characterization of primary mouse aortic endothelial cells (MAECs). **a** Aortic segments cultured in Matrigel demonstrated the ability to form tubular networks and can be used for EC isolation. Scale bar: 50 µm. **b** isolated MAECs in culture (see methods). Scale bar: 100 µm. **c** Comparison of Vascular endothelial growth factor receptor 2 (VEGFR2) expression in isolated MAECs and mouse brain endothelial cells (MBECs). **d** quantification of the representative data from (**b**) (n=7 independent experiments; data was assessed using Student's t-test and is presented as the mean ± SEM. *P<0.0001). **e, f** Immunofluorescent staining of the endothelial markers von Willebrand factor (vWF) (**e**) and CD31 (**f**) with alpha smooth muscle actin (α-SMA) in isolated MAECs. Repeated three times. Scale bars 200 µm. **g** Weibel–Palade bodies were revealed by vWF staining at high magnification. Scale bar 10 µm. **h** Epsin 1 and 2 deletion in MAECs induced by 5 µM tamoxifen for 4 days in culture was confirmed by western blot analysis for epsin 1, epsin 2, and tubulin. This is a representative image of at least five independent repeats.

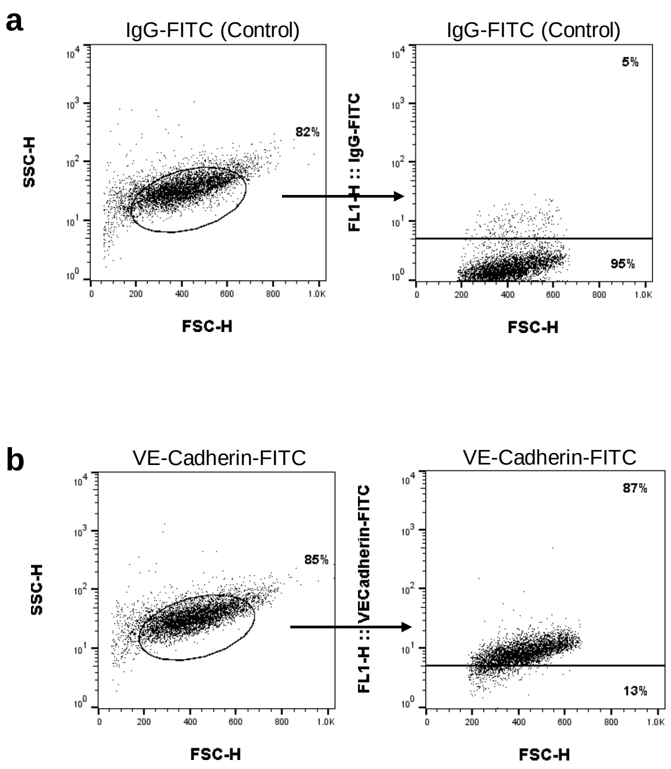

Supplementary Figure 3 Characterization of MAECs by flow cytometry using an isotype-specific control (left) or VE-cadherin antibody (right) with gating strategy to determine the percentage of MAECs expressing endothelial marker VE-Cadherin. n=5 replicates.

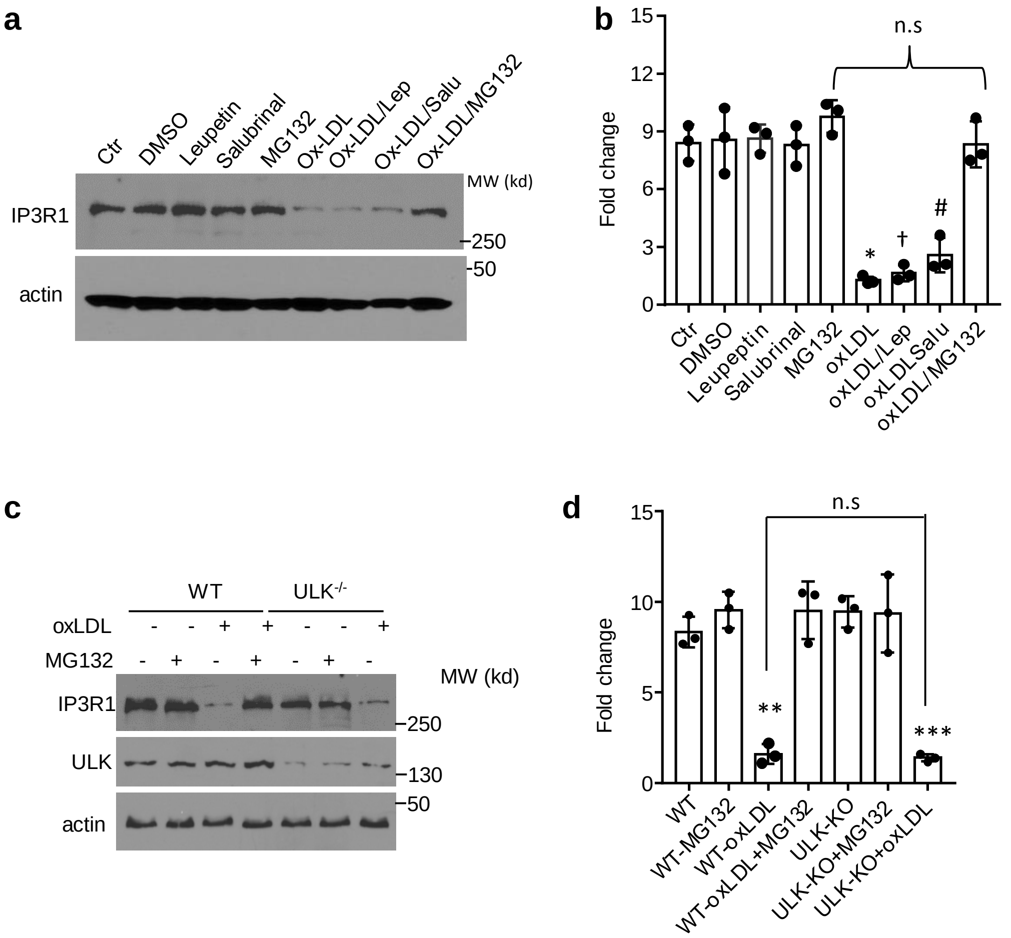

Supplementary Figure 4 Test if IP3R1 can be degraded by other protein degradation system. Lysosome, UPR and autophagy were examined using biochemical inhibitors (lysosome and UPR) or genetic inhibition approach (autophagy).We repeated three times for each experiment. (**a**, **b**) 100 μM Leupeptin, a lysosomal inhibitor or 10 μM Salubrinal, an UPR inhibitor can not reverse IP3R1 degradation by oxLDL treatment (36 hours); (**c**, **d**) Genetic deletion of ULK, a gene in autophagy pathway, cannot reverse the IP3R1 degradation by 100 μg/ml oxLDL. Proteasomal inhibitor MG132 served as positive control (at 2.5 μM). Quantification (b, d) was performed from three independent experiments. All data were assessed using Student's t-test and are presented as the mean ± SEM. n=3. In (**b**), n=3, *P<0.01, Ctr vs oxLDL; +P<0.01, Lep vs oxLDL/Lep, #P<0.01, Salu vs oxLDL/Salu; n.s, no statistical difference, MG132 vs oxLDL/MG132. In (**d**), n=3, ** P<0.01, WT-MG132 vs WT-oxLDL/MG132; *** P<0.01, ULK-KO vs ULK-KO/oxLDL; n.s: no statistical difference, WT/oxLDL vs ULK-KO/oxLDL. All data were assessed using Student's t-test and are presented as the mean ± SEM.

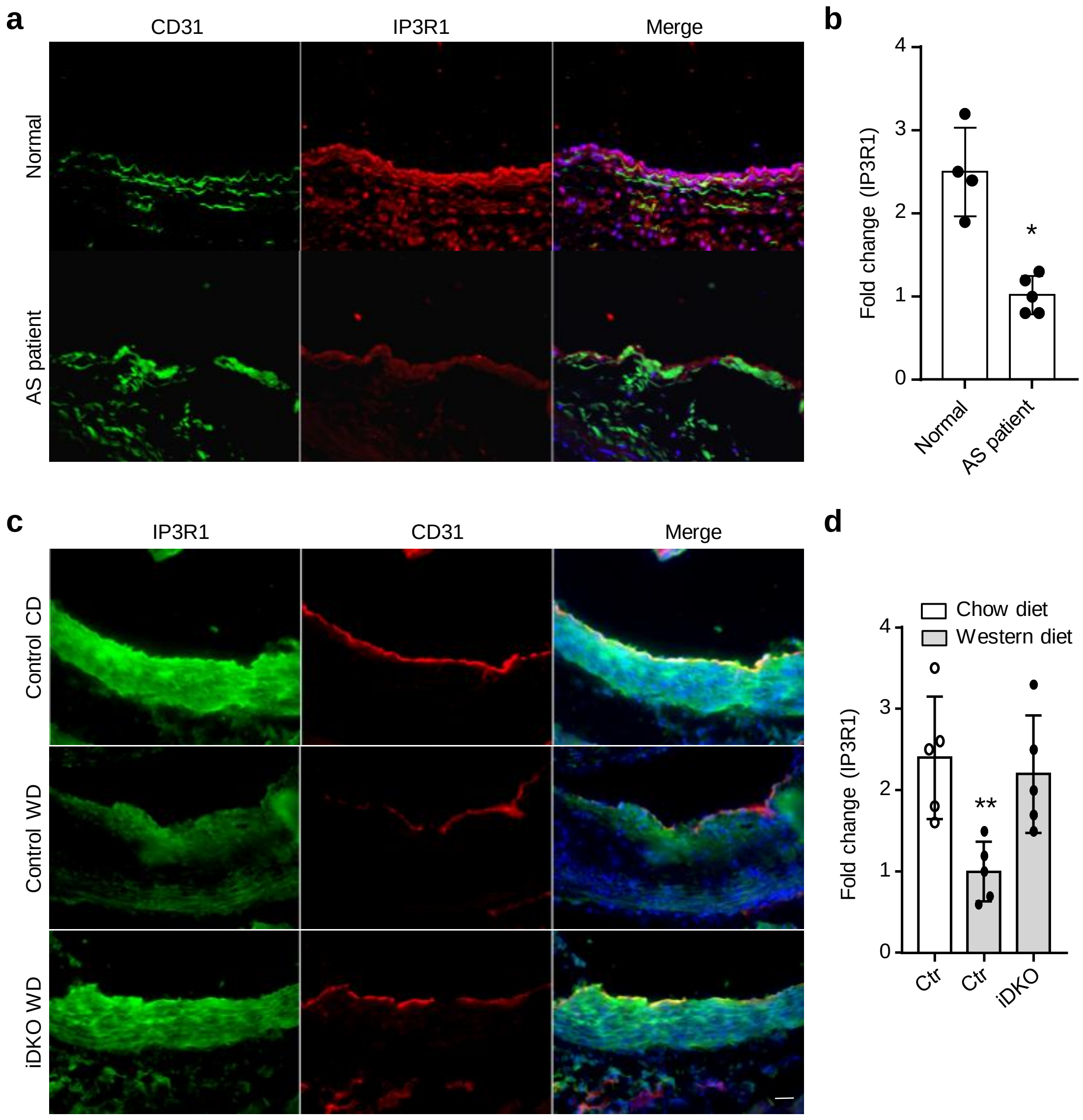

Supplementary Figure 5 Epsins regulate IP3R1 stability in human atherosclerosis (AS) patients and mice. **a, b** Immunofluorescent staining of IP3R1 (red) and CD31 (green) in atherosclerotic patient aortas versus healthy controls (n=5 in each group) (**a**). Quantification of IP3R1 immunostaining data in the endothelium (n=5, *P<0.01) (**b**). Scale bar 100 µm. **c, d** Immunofluorescent staining of IP3R1 (green) and CD31 (red) in the aortas of control (ApoE$^{-/-}$) (middle panels) and iDKO (EC-iDKO/ApoE$^{-/-}$) (lower panels) mice fed a western diet (WD) (n=5 in each group). ApoE$^{-/-}$ mice fed a regular chow diet (CD) were similarly immunostained for comparison (top panels) (**c**). IP3R1 immunostaining in the endothelium was quantified (n=5, *P<0.01 between control groups) (**d**). Scale bar: 100 µm. All data were assessed using Student's t-test and are presented as the mean ± SEM. *P<0.05, compared to normal people; n=5, ** P<0.05, CD Ctr vs WD Ctr, and WD Ctr vs WD EC-iDKO. All data were assessed using Student's t-test and are presented as the mean ± SEM.

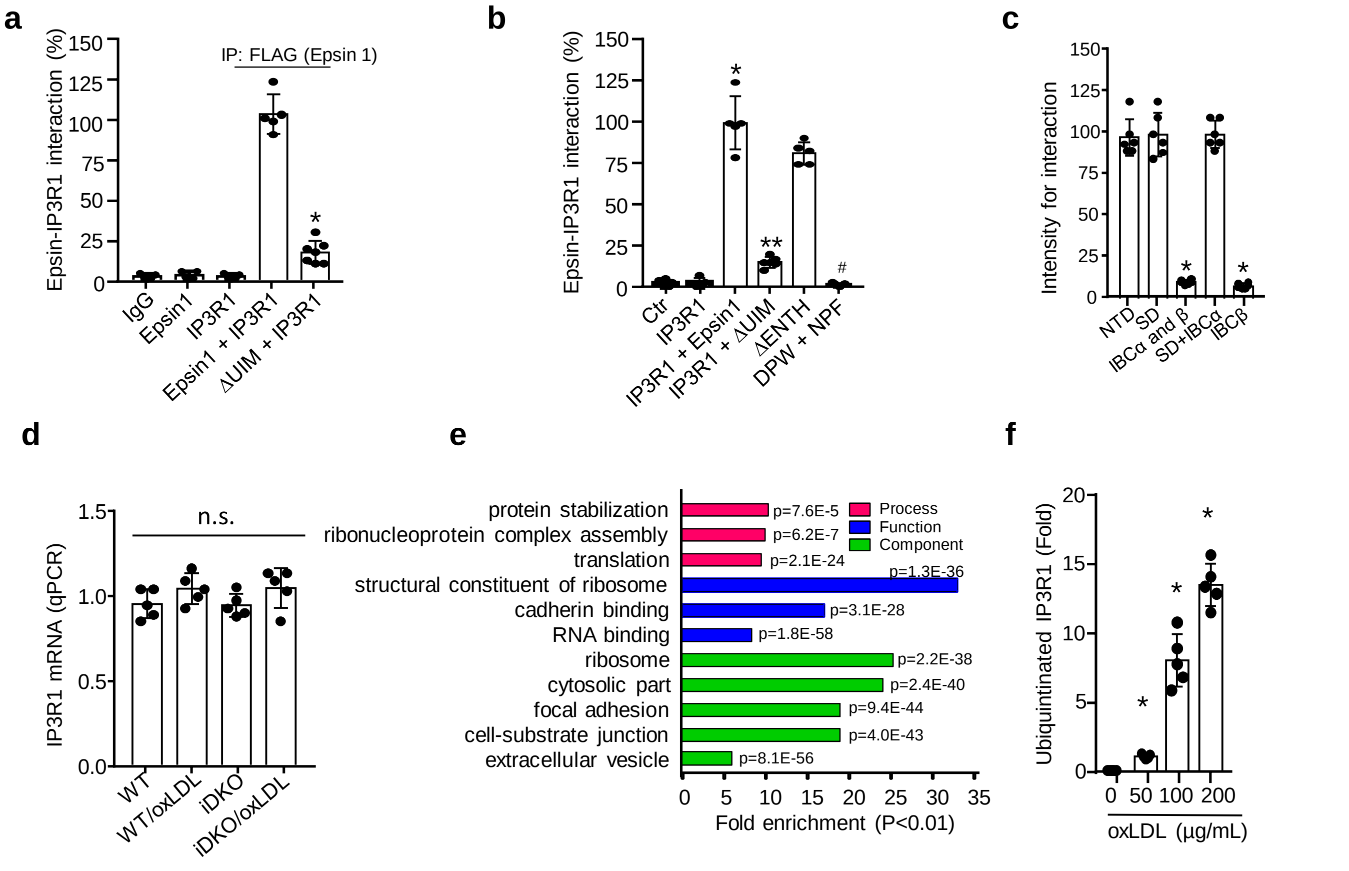

Supplementary Figure 6 Data quantification from Figures, IP3R1 mRNA levels, and gene ontology analysis. **a** to **c** Data quantification from Figs. 2B, 2C, and 2H. (**a** to **c**, n=6-7 independent repeats as indicated for each experiment, *P<0.001). **d** IP3R1 mRNA levels in WT ($ApoE^{-/-}$) and iDKO MAECs in response to 100 µg/mL oxLDL treatment for 36 h. **e** The most significant gene ontology (GO) analyses on epsin 1 binding proteins reveal that epsin 1 is involved in controlling protein stabilization. P values are indicated in the histogram. **f** Data quantification from Fig. 3C. n=5 independent repeats. In (a), *P<0.001, Epsin 1+IP3R1 vs ΔUIM+IP3R1; in (b), *P<0.001, Ctr or IP3R1 vs IP3R1+Epsin 1; ** P<0.001, IP3R1+Epsin 1 vs IP3R1+ΔUIM; # P<0.001, IP3R1+Epsin 1 vs IP3R1+DPW+NPF; in (c), *P<0.001, IBCα+β, or IBCβ vs NTD or SD; in (d), n.s, no statistical difference among groups; in (f), n=5, *P<0.001, different concentration of oxLDL compared to control. All data were assessed using Student's t-test and are presented as the mean ± SEM.

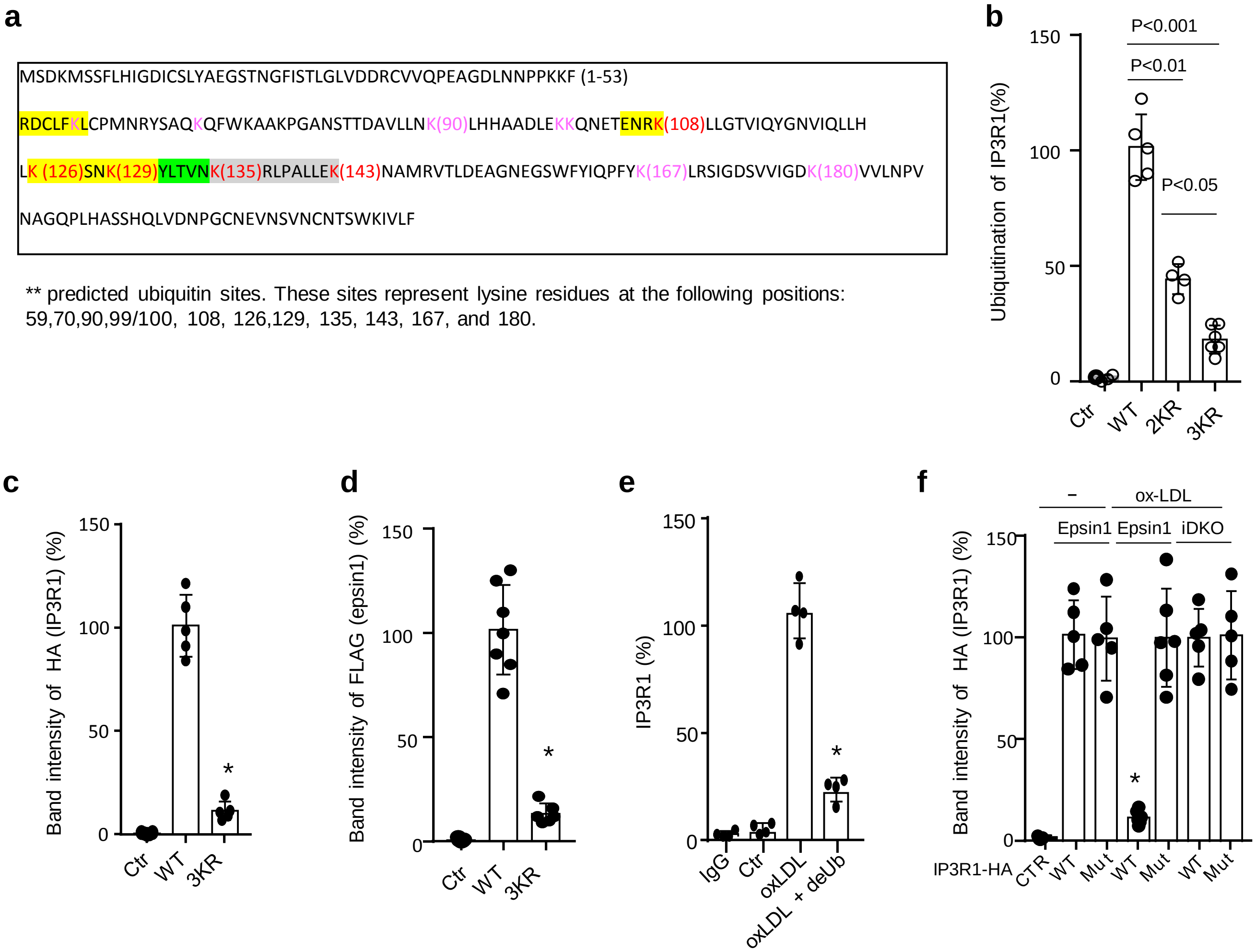

Supplementary Figure 7 Predicted ubiquitination sites and data quantification from figures. **a** Prediction of the putative ubiquitin sites are indicated for amino acids (aa) in the SD of IP3R1. **b** to **e** Data quantification for Figures 3D to G. **f** Data quantification for Fig. 3L. For these *in vitro* experiments n=5 in (**b**), n=5 in (**c**), n=7 in (**d**), n=4 in (**e**) and n=6 in (**f**); *P<0.001. In (b), P<0.01 WT vs 2KR; P<0.001 WT vs 3KR; in (c) *P<0.001, WT vs 3KR; in (d), *P<0.001 WT vs 3KR; in (e), oxLDL vs oxLDL+deUb; in (f), *P<0.001, WT vs WT/oxLDL, however, Epsin 1 mutant or Epsin 1 KO will not cause IP3R1 degradation under oxLDL treatment. All data were assessed using Student's t-test and are presented as the mean ± SEM.

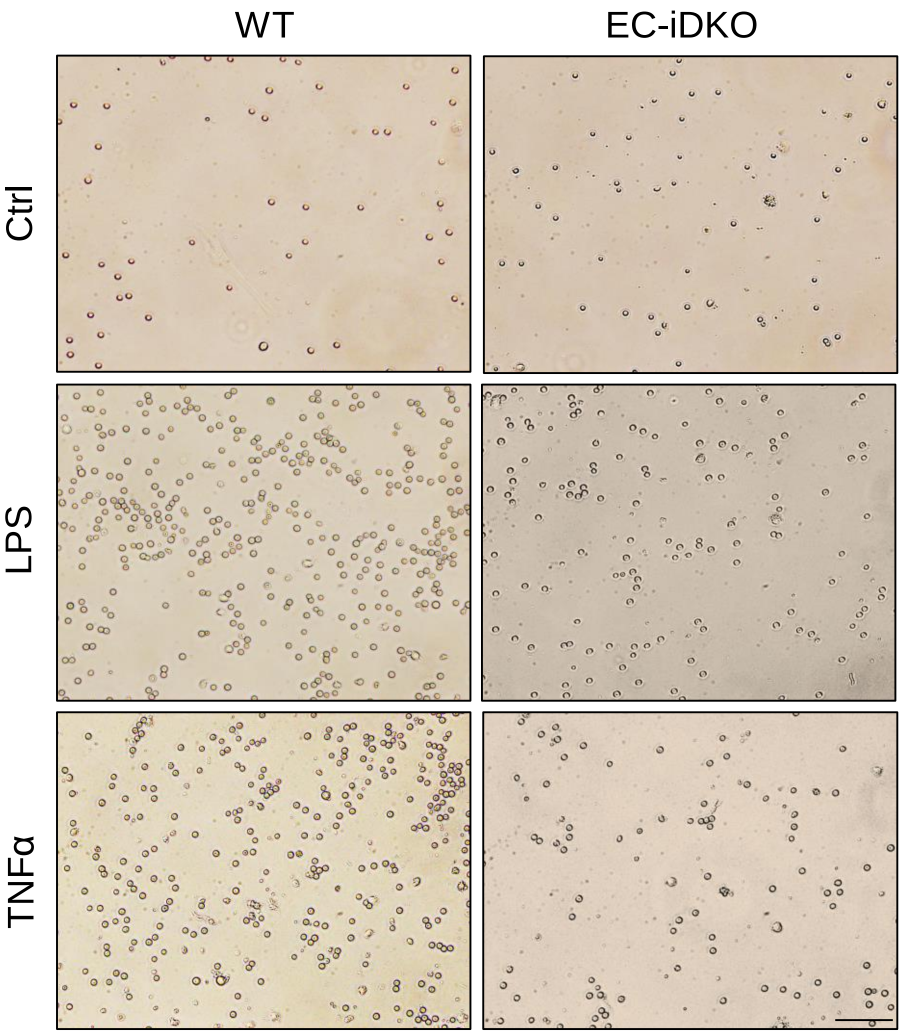

Supplementary Figure 8 Macrophage migration assay onto MAECs of WT and EC-iDKO, showing representative images. MAECs of WT and EC-iDKO were isolated from mice and epsin 1 and 2 were deleted in culture dishes for 4 days. MAECs then were transferred to migration transwell to culture overnight. MAECs were then stimulated with LPS (200 ng/ml) or TNFα (50 ng/ml) for 3 hours. Mouse macrophages were isolated from WT mice and added onto the top of transwell, allowing macrophage to penetrate and migrate for 3 hours. Migrated macrophages attached to the plate bottom and recorded under microscopy. Repeated 3 times. Nine fields were captured for each treatment for cell count and statistical analysis. Scale bar: 100 μm.6

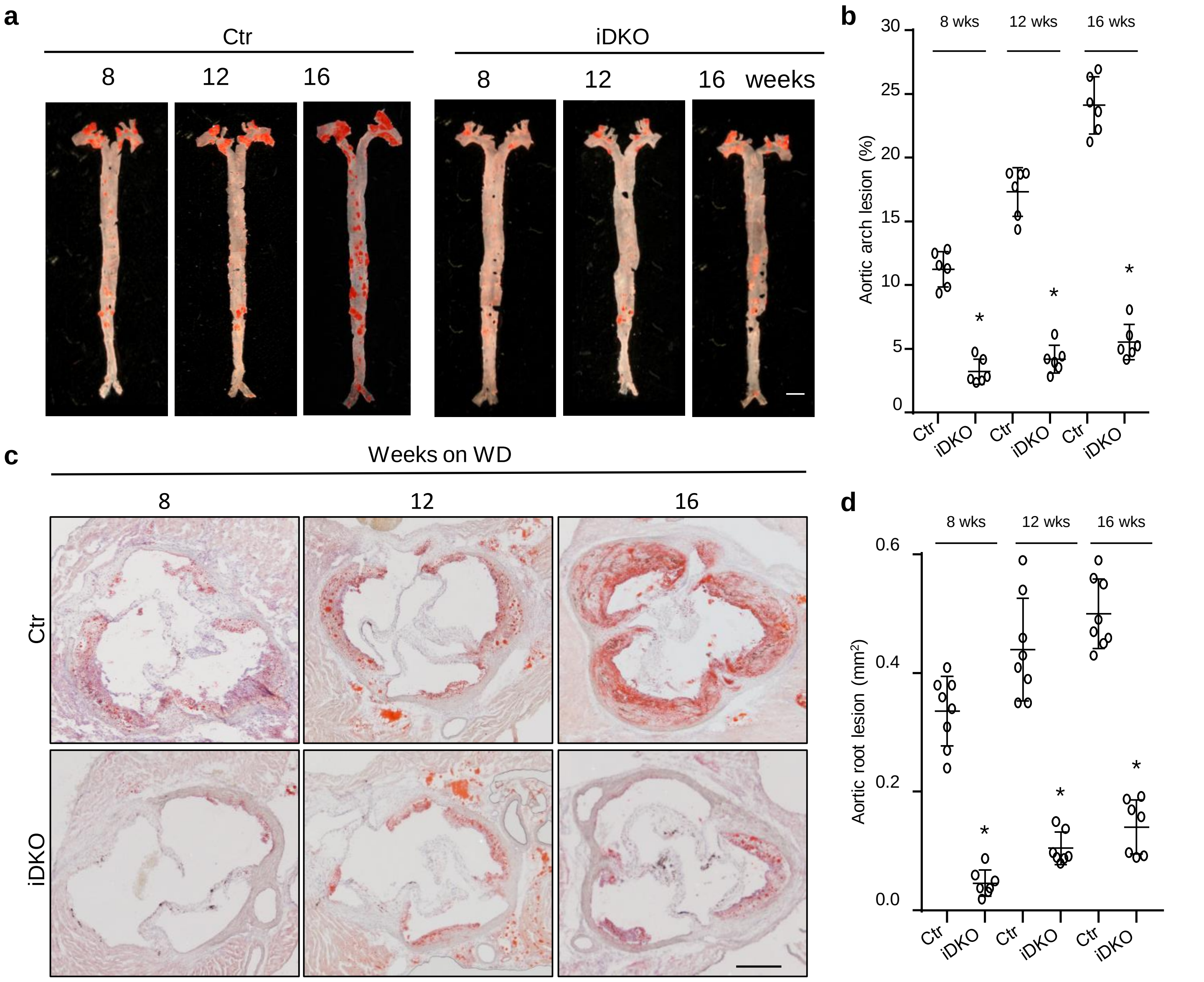

Supplementary Figure 9 Analysis of control and iDKO mice by Oil Red O staining. **a** Eight-week-old mice were fed a WD for the indicated times and then aortic arches were stained with ORO. **b** Data quantification for (**a**) (n=6 mice in each group, *P<0.001). **c** Eight-week-old mice were fed a WD for the indicated times and then aortic roots were stained with ORO. **d** Data quantification for (**c**) (n=8 wt mice were analyzed in 12 and 16 weeks; in all other groups, seven mice were analyzed. *P<0.001). Ctr vs iDKO in different time point. All data were assessed using Student's t-test and are presented as the mean ± SEM. Scale bars, 5 mm for (a), 500 μm for (b).

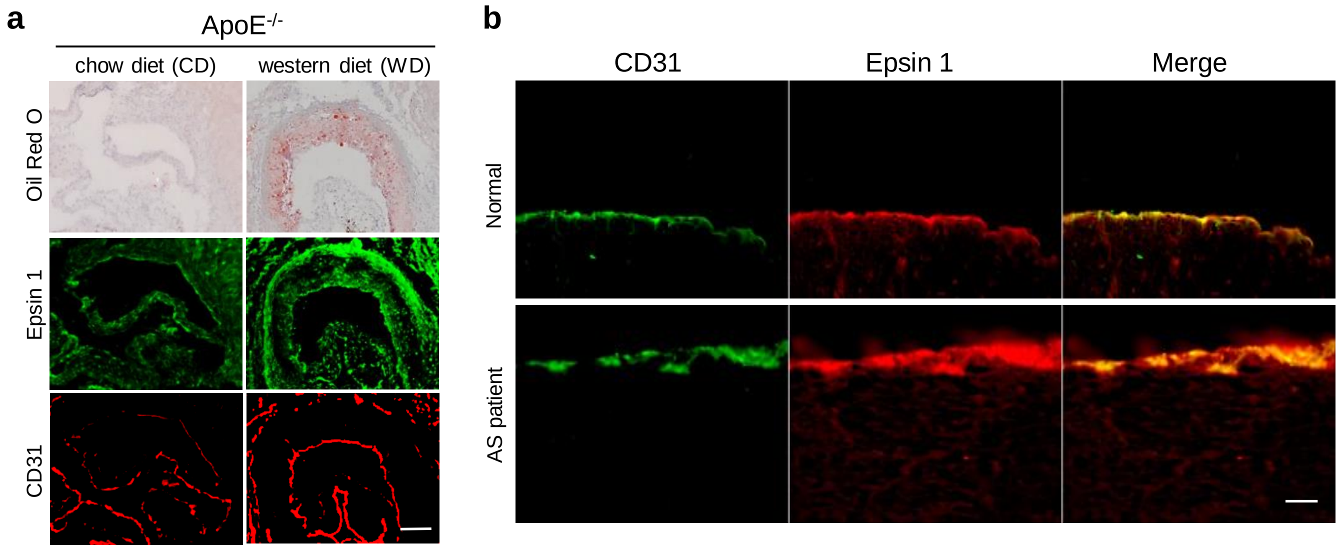

Supplementary Figure 10 Epsins are upregulated in atherosclerotic mice and human atherosclerotic patients. **a** Epsin 1 expression in aortic roots by immunofluorescent staining using epsin 1 antibody and co-staining with CD31 (EC marker) in ApoE$^{-/-}$ mice fed a regular chow diet (CD) or western diet (WD) for 10 weeks. n=3. Scale bar 500 µm. **b** CD31 and epsin 1 immunostaining of human normal and atherosclerotic aortic arch samples, n=4 for normal samples, and n=5 for atherosclerotic aortic arch samples. Scale bar 100 µm.

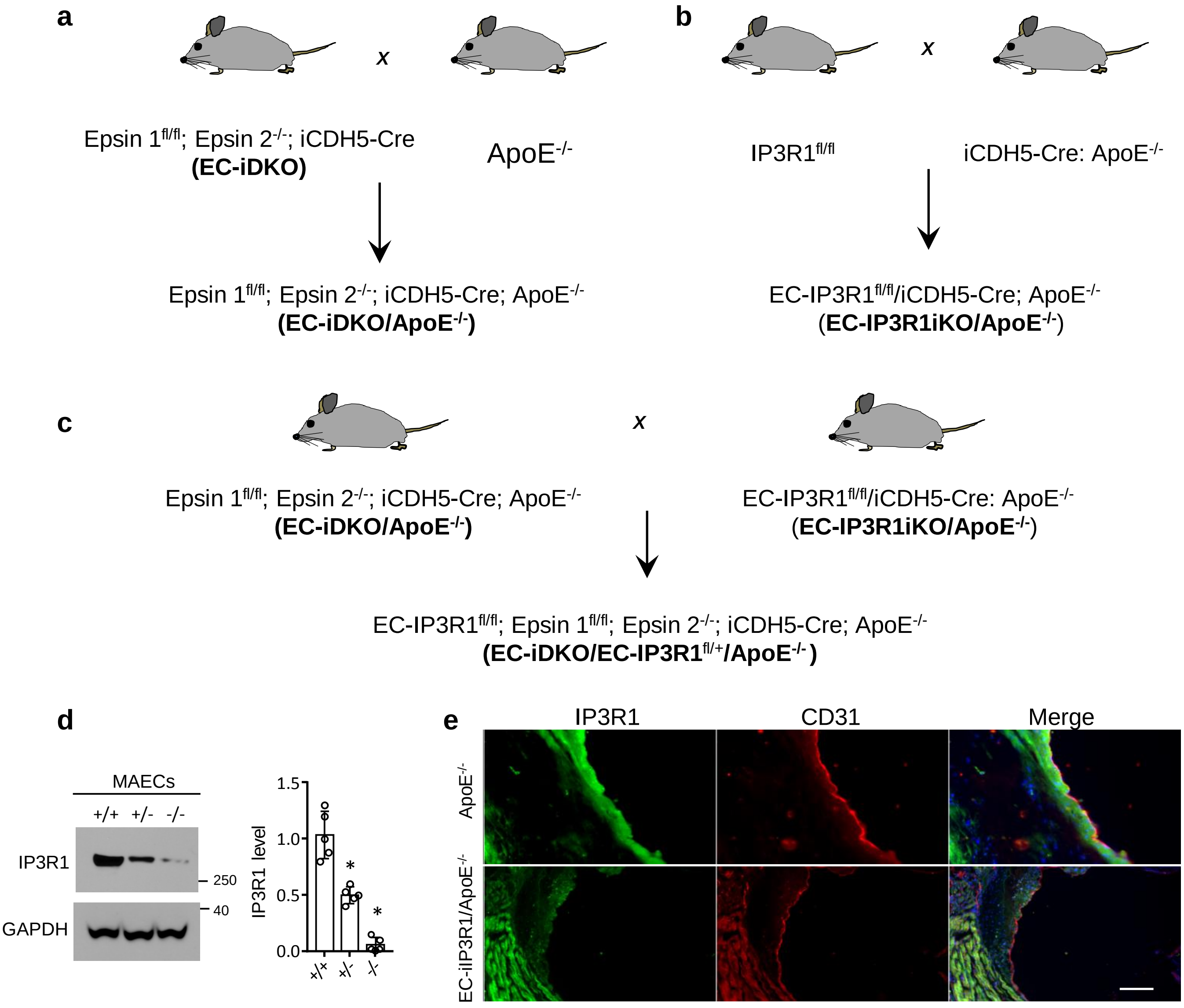

Supplementary Figure 11 Strategy for generating EC-IP3R1iKO/ApoE$^{-/-}$ and EC-IP3R1iKO/iDKO/ApoE$^{-/-}$ or EC-IP3R1$^{fl/+}$/iDKO/ApoE$^{-/-}$ mice. **a** Generation of EC-iDKO/ApoE$^{-/-}$ mice. **b** Generation of EC-IP3R1iKO/ApoE$^{-/-}$ mice. **c** Generation of EC-IP3R1$^{fl/+}$/iDKO/ApoE$^{-/-}$ mice. **d, e** IP3R1 expression in MAECs isolated from ApoE$^{-/-}$, EC-IP3R1$^{fl/+}$:iCDH5-Cre ERT2, and EC-IP3R1$^{fl/fl}$:iCDH5-Cre ERT2 mice (+/+, +/- and -/-) after 4-hydroxytamoxifen treatment assessed using western blot analysis. n=5 independent experiments from different MAECs; *P<0.001. (**d**) and in the endothelium of ApoE$^{-/-}$ (upper panels) and EC-IP3R1$^{fl/fl}$:iCDH5-Cre mice (lower panels) evaluated by immunofluorescent staining using anti-IP3R1 and CD31 antibodies (**e**). Data was assessed using Student's t-test and is presented as the mean ± SEM. *P<0.001, +/+ vs +/- or -/-. Scale bar: 100 μm.

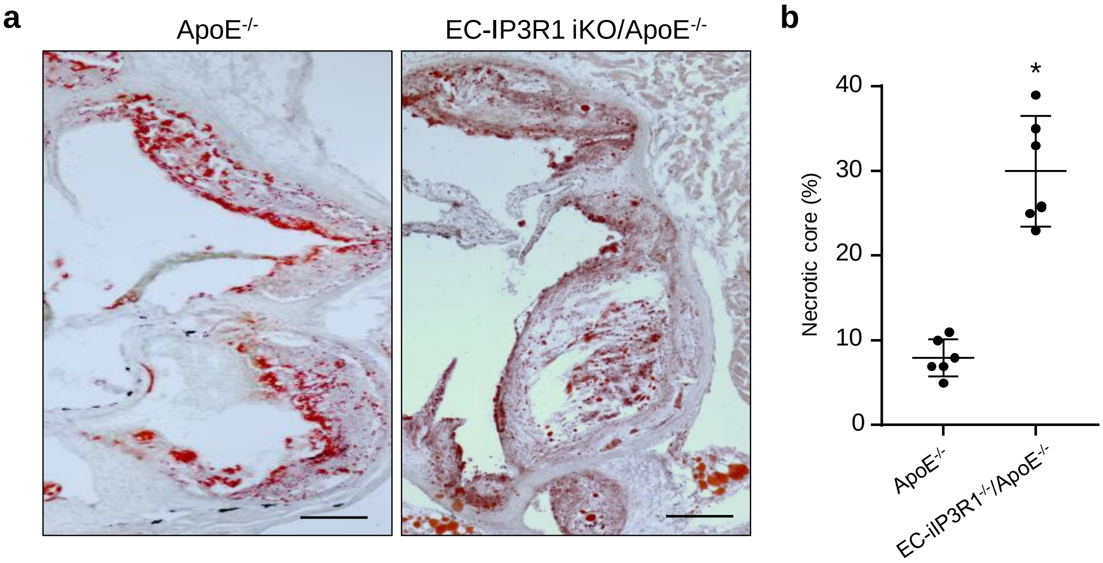

Supplementary Figure 12 EC-IP3R1iKO/ApoE$^{-/-}$ mice exhibit significant necrotic cores. **a**, **b** Necrotic cores (%) in ApoE$^{-/-}$ and EC-IP3R1iKO/ApoE$^{-/-}$ mice fed a WD for 25 weeks (**a**) and quantification of core size (**b**) (n=6, *P<0.05). Data was assessed using Student's t-test and is presented as the mean ± SEM. * P<0.001, ApoE$^{-/-}$ vs ApoE$^{-/-}$/EC-IP3R1$^{-/-}$. Scale bar: 500 µm.
