# Supplementary material for: Epsin-mediated degradation of IP3R1 fuels atherosclerosis
Source: Nat Commun. 2020 Aug 7;11:3984. doi: 10.1038/s41467-020-17848-4 (PMC7414107; doi:10.1038/s41467-020-17848-4)
Supplement: Supplementary file 5 — Supplementary Data 4 [file 41467_2020_17848_MOESM5_ESM.docx]

**Supplementary Data file 4**

| REAGENT or RESOURCE | SOURCE | Note |
| --- | --- | --- |
| Antibodies | | |
| Goat polyclonal anti-Epsin1 | Santa Cruz Biotechnology | Cat# sc-8673; RRID: AB_2231153 |
| Rabbit polyclonal anti-Epsin1 | Home made | N/A |
| Rabbit polyclonal anti-Epsin2 | abcam | Cat# ab74942; RRID: AB_2231155 |
| Rabbit polyclonal anti-Epsin2 | Home made | N/A |
| Rabbit polyclonal anti-IP3R1 | Santa Cruz Biotechnology | Cat# sc-28614; RRID: AB_2296450 |
| Macrophages / Monocytes Monoclonal Antibody (MOMA-2) | Thermo Fisher Scientific | Cat# MA1-80630, RRID:AB_934726 |
| Rat monoclonal anti-F4/80 | Santa Cruz Biotechnology | Cat# sc-59171, RRID:AB_1122716 |
| Rat-anti Mouse CD31 | BD Pharmingen | Cat# 550274;RRID: AB_393571 |
| Rabbit polyclonal anti-vWF | abcam | Cat# ab6994, RRID:AB_305689 |
| Monoclonal anti-HA.11 (Western blot) | BioLegend | Cat# 901502; RRID: AB_901502 |
| Polyclonal anti-HA antibody (IP) | Clontech | Cat# 631207; RRID:N/A |
| Monoclonal anti-Flag | Sigma-Aldrich | Cat# F3165; RRID: AB_259529 |
| Rabbit monoclonal anti-Phosphor-PERK | Cell Signaling Technology | Cat# 3179S, RRID:AB_2095853 |
| Rabbit polyclonal anti-PERK | Santa Cruz Biotechnology | Cat# sc-13073;RRID: AB_2230863 |
| Rabbit monoclonal Phosphor-eIF2α | Cell Signaling Technology | Cat# 3398, RRID:AB_2096481 |
| Rabbit monoclonal eIF2α | Cell Signaling Technology | Cat# 5324, RRID:AB_10692650 |
| Rabbit monoclonal Phosphor-SAPK/JNK | Cell Signaling Technology | Cat# 4668S, RRID:AB_823588 |
| Rabbit polyclonal SAPK/JNK Antibody | Cell Signaling Technology | Cat# 9252, RRID:AB_2250373 |
| Rabbit polyclonal anti-KDEL | StressMarq Biosciences | Cat# SPC-109B, RRID:AB_10805031 |
| Monoclonal anti-ATF6 | Santa Cruz Biotechnology | Cat# sc-166659; RRID: AB_2058901 |
| Rabbit polyclonal anti-XBP1 | Santa Cruz Biotechnology | Cat# sc-7160, RRID:AB_794171 |
| Goat polyclonal anti-GRP78 | Santa Cruz Biotechnology | Cat# sc-1051, RRID:AB_2119994) |
| Rabbit polyclonal antibody anti-p38 | Cell Signaling Technology | Cat# 9212, RRID:AB_330713 |
| Rabbit polyclonal Anti-p38 MAPK, phosphor (Thr180 / Tyr182) Antibody | Cell Signaling Technology | Cat# 9211, RRID:AB_331641 |
| Rabbit polyclonal Anti-RPN2 antibody | Sigma-Aldrich | Cat# HPA025922, RRID:AB_1856458 |
| Rabbit polyclonal anti-Ribosomal Protein S3 | Cell Signaling Technology | Cat# 2579S, RRID:AB_2182395 |
| Mouse monoclonal anti-ubiquitin | Cytoskeleton, Inc | Cat# AUB01; RRID:N/A |
| Rabbit monoclonal anti-NF-kB | Cell Signaling Technology | Cat# 8242, RRID:AB_10859369 |
| Phospho-NF-κB p65 (Ser536) (93H1) Rabbit mAb | Cell Signaling Technology | Cat# 3033, RRID:AB_331284 |
| Rabbit polyclonal anti-ABCA1 | Novus Biologicals | NB400-105SS; RRID:AB_535486 |
| Rabbit polyclonal anti-ABCG1 | Novus Biologicals | NB400-132B, RRID:AB_1216142 |
| Goat polyclonal anti mouse LDLR | R & D System | Cat# AF2255, RRID:AB_355203 |
| Rat monoclonal Anti-mouse Lox-1 | R & D System | Cat# MAB1564, RRID:AB_2157691 |
| Rat monoclonal Anti-mouse /SR-B3CD36 | R & D System | Cat# MAB2519, RRID:AB_2072634 |
| Rat monoclonal Anti-mouse SRA1 | R & D System | Cat# MAB1797, RRID:AB_2148245 |
| Goat polyclonal anti-SRB1 | Novus Biologicals | NB400-131SS; RRID:AB_792009 |
| Anti-Actin, α-Smooth Muscle - Cy3™ antibody, Mouse monoclonal α-SMA | Sigma-Aldrich | Cat# C6198, RRID:AB_476856) |
| Rabbit monoclonal anti-VEGFR2 | Cell Signaling Technology | Cat# 2479L, RRID:AB_2212507) |
| CD45-FITC (FACS) | eBioscience | Cat# 8011-945-120; RRID:N/A |
| Rat monoclonal, PerCP anti mouse CD45 (FACS) | BioLegend | Cat# 103129, RRID:AB_893343 |
| Rabbit polyclonal anti-VECadherin-FITC (FACS) | AbD Serotec | Cat# AHP628F, RRID:AB_2077974 |
| PE/Cy7 anti-mouse CD11b (FACS) | BioLegend | Cat# 101216, RRID:AB_312799 |
| Hamster monoclonal antibody, CD11c (FACS) | BioLegend | Cat# 117301, RRID:AB_313770 |
| Rat monoclonal PE anti-mouse CD19 (FACS) | BioLegend | Cat# 115507, RRID:AB_313642 |
| Alexa Fluor® 488 anti-mouse TCR β chain Antibody TcRβ (FACS) | BioLegend | Cat# 109216, RRID:AB_493345 |
| Rat monoclonal Pacific Blue anti-mouse-LyC6 (FACS) | BioLegend | Cat# 128013, RRID:AB_1732090 |
|  |  |  |
| Bacterial and Virus Strains | | |
| NEB® 5-alpha Competent E. coli (High Efficiency): cloning | New England Biolabs | Cat# C2987P |
| XL10-Gold Ultracompetent Cells: cloning | Agilent Technologies | Cat# 200315 |
|  |  |  |
| Biological Samples |  |  |
| Atherosclerosis patient’s arch samples | Maine Medical Center (MRC) BioBank, Maine | N/A |
| Normal population’s arch samples | Maine Medical Center (MRC) BioBank, Maine | N/A |
| Mouse aortic roots and hearts | This study | N/A |
| Mouse serum | This study | N/A |
| Chemicals, Peptides, and Recombinant Proteins | | |
| Oil Red O (ORO) | AMRESCO | Cat# 0684-100G |
| Tamoxifen | Sigma-Aldrich | Cat# T5648-1G |
| ox-LDL | Dong et al. 2010b | N/A |
| Cholesterol | Sigma-Aldrich | Cat# C8667 |
| 7-ketocholesterol (7-KC) | Sigma-Aldrich | Cat# C2394 |
| N-Ethylmaleimide (NEM) | Alfa Aesar | Cat# 40526 |
| MG132 | Sigma-Aldrich | Cat# M7449 |
|  |  |  |
| Critical Commercial Assays | | |
| Glucose measurement - OneTouch Glucometer | LifeScan | Cat#:  LIF021-098-EA |
| HDL and LDL/VLDL Cholesterol Assay Kit | abcam | Cat# ab65390 |
| Triglyceride Quantification Kit | abcam | Cat# ab65336 |
| BAC-protein concentration measurement kit | Thermo Fisher Scientific | Cat# 23225 |
| SuperSignal™ West Pico Chemiluminescent Substrate | Thermo Fisher Scientific | Cat#: 34080 |
|  |  |  |
| Deposited Data | | |
| N/A |  |  |
|  |  |  |
| Experimental Models: Cell Lines | | |
| Primary cultured mouse aortic endothelial cells (MAECs) | This study | N/A |
| 293T | ATCC | CRL-3216 |
|  |  |  |
| Experimental Models: Organisms/Strains | | |
| ApoE^-/-^ atherosclerosis mouse model  (B6.129-Apoe^tm1Unc^) | Jackson Laboratory | Cat# 002052 |
| Epsin mutant mouse model | This study | N/A |
| IP3R1 mutant mouse model | This study | N/A |
| Carotid atherosclerosis mouse model | This study | N/A |
| iCDH5-Cre mouse strain (B6;129-Tg(Cdh5-cre)1Spe/J) | Jackson Laboratory | Stock No: 017968 |
|  |  |  |
| Oligonucleotides | | |
| N🡪Q point mutation (N24Q)  Forward: 5-gtatgcggagggatctacgcagggatttatcagcaccttag  Reverse: 5-ctaaggtgctgataaatccctgcgtagatccctccgcatac | This study | N/A |
| N24 deletion:  Forward: 5-gtatgcggagggatctacgggatttatcagcaccttag  Reverse: 5-ctaaggtgctgataaatcccgtagatccctccgcatac | This study | N/A |
| NGF deletion:  Forward: 5-gtatgcggagggatctacgatcagcaccttaggcttgg  Reverse: 5-ccaagcctaaggtgctgatcgtagatccctccgcatac | This study | N/A |
| NTD  Forward: 5-agaattcatgtctgacaaaatgtcgagtttcc  Reverse: 5-tctcgagttatttccggttgttgtggagcagggc | This study | N/A |
| SD  Forward: 5-agaattcatgtctgacaaaatgtcgagtttcc  Reverse: 5-tctcgagttagaaaagcactatcttccagcttgt | This study | N/A |
| IBC:  Forward: 5- agaattcttcatgaaatggagtgataacaaag  Reverse: 5- tctcgagttatttccggttgttgtggagcagggc | This study | N/A |
| SD+α  Forward: 5-agaattcatgtctgacaaaatgtcgagtttcc  Reverse: 5- tctcgagttagtccaggtcccgaacctcagcagg | This study | N/A |
| Beta (β) of NTD  Forward: 5-agaattcgactttgccaatgatgccagcaaggtgctg  Reverse: 5-tctcgagttatttccggttgttgtggagcagggc | This study | N/A |
| IP3R1∆53 (Infusion cloning to pCDNA3.1)  Forward: 5-agcacagtggcggccgcagagactgcctctttaagc  Reverse: 5-aaacgggccctctagactaggccggctgctgtgg | This study | N/A |
| K126, 129 (K to R)  Forward: 5-ctcctgcatttgagaagcaatagatacctgactgtg  Reverse: 5-cacagtcaggtatctattgcttctcaaatgcaggag | This study | N/A |
| K143 (K to R)  Forward: 5-ccagccttgctagagaggaacgccatgagggtgac  Reverse: 5-gtcaccctcatggcgttcctctctagcaaggctgg | This study | N/A |
| ICAM-1: | This study | N/A |
| Forward: 5- gtg gtg aag tct gt caa aca gga g  Reverse: 5- cct cct gag cct tct gta act tgt |  |  |
| VCAM-1: | This study | N/A |
| Forward: 5- ctc tcc cag gaa tac aac gat ctc |  |  |
| Reverse: 5- gac tcc aga gtc ttc cat cct cat |  |  |
| P-selectin | This study | N/A |
| Forward: 5- atc cag gaa gct ctg acg tac ttg  Reverse: 5- cag cgt tag tga aga ctc cgt atg |  |  |
| E-selectin | This study | N/A |
| Forward: 5- gtt tga ctg tgt gga agg gta cag  Reverse: 5- gct cac agg tga agt tac agg atg |  |  |
| MCP-1 | This study | N/A |
| Forward: 5- cca ctc acc tgc tgc tac tca  Reverse: 5- tgg tga tcc tct tgt agc tct cc |  |  |
| INF-gamma | This study | N/A |
| Forward: 5- aag tgg cat aga tgt gga aga aa  Reverse: 5- ttg acc tca aac ttg gca ata ct |  |  |
| TNF-alpha | This study | N/A |
| Forward: 5- acg gca tgg atc tca aag ac  Reverse: 5- aga tag caa atc ggc tga cg |  |  |
| IL-6 | This study | N/A |
| Forward: 5- ctg gag tac cat agc tac ctg ga  Reverse: 5- ctt agc cac tcc ttc tgt gac tc |  |  |
| IL-1beta | This study | N/A |
| Forward: 5- tac aag gag aac caa gca acg a  Reverse: 5- gct tgt gag gtg ctg atg tac c |  |  |
| IL-10 | This study | N/A |
| Forward: 5- gcc aca tgc tcc tag agc tg  Reverse: 5- cag ctg gtc ctt tgt ttg aaa |  |  |
| GAPDH | This study | N/A |
| Forward: 5- ctc atg acc aca gtc cat gc  Reverse: 5- cac att ggg ggt agg aac ac |  |  |
| Beta-actin | This study | N/A |
| Forward: 5- gat caa gat cat tgc tcc tcc tg  Reverse: 5- agg gtg taa aac gca gct ca |  |  |
| Human IP3R1 flow | This study | N/A |
| Forward: 5- tgcctcagtgagaaagagca  Reverse: 5- aagcttgattctgctggttg |  |  |
| Human actin control: | This study | N/A |
| Forward: 5- gagcacagagcctcgccttt |  |  |
| Reverse: 5- tcatcatccatggtgagctgg |  |  |
|  |  |  |
| Recombinant DNA | | |
| Epsin1^WT.HA^ | Pasula et al, 2012 | N/A |
| Epsin1^WT.Flag^ | Chang et al, 2015 | N/A |
| Epsin1^∆UIM.HA^ | Pasula et al, 2012 | N/A |
| Epsin1^∆ENTH.HA^ | Chang et al, 2015 | N/A |
| Epsin1^∆ENTH+UIM.HA^ (=DPW+NPF) | Chang et al, 2015 | N/A |
| IP3R1HA^WT^ | Zhu et al, 1999 | N/A |
| IP3R1HA^∆1-1581^ | Zhu et al, 1999 | N/A |
| IP3R1HA^∆1-1903^ | Zhu et al, 1999 | N/A |
| IP3R1HA^∆1-2268^ | Zhu et al, 1999 | N/A |
| IP3R1HA^∆53^ | This study | N/A |
| NTD+RD | This study | N/A |
| RD | This study | N/A |
| NTD | This study | N/A |
| NTD^∆53^ | This study | N/A |
| SD | This study | N/A |
| SD^∆53^ | This study | N/A |
| IBC | This study | N/A |
| SD+α | This study | N/A |
| SD+α^∆53^ | This study | N/A |
| Βeta (β) of NTD | This study | N/A |
| IP3R1^dub^ (K126/129 double ub mutations) | This study | N/A |
| IP3R1^tub^ (K126/129/143 triple ub mutations) | This study | N/A |
|  |  |  |
| Software and Algorithms | | |
| Image J | NIH website: <https://imagej.nih.gov/ij/> | N/A |
| GraphPad Prism | GraphPad software company: <https://www.graphpad.com/scientific-software/prism/> | N/A |
| Gene ontology (GO) analysis | GO term finder http://go.princeton.edu/ | N/A |
| switches.ELM.eu.org | http://switches.elm.eu.org/index.php | N/A |
|  |  |  |
| Other | | |
| rec-Protein G-Sepharose® 4B beads | Life Technologies | Cat# 101242 |
| Anti-FLAG® M2 Affinity Gel | Sigma-Aldrich | Cat# A2220 |
