# Supplementary material for: Epsin-mediated degradation of IP3R1 fuels atherosclerosis
Source: Nat Commun. 2020 Aug 7;11:3984. doi: 10.1038/s41467-020-17848-4 (PMC7414107; doi:10.1038/s41467-020-17848-4)
Supplement: Supplementary file 6 — Reporting Summary [file 41467_2020_17848_MOESM6_ESM.pdf]

nature research

Corresponding author(s): Hong Chen

Last updated by author(s): 6/16/2020

Reporting Summary

Nature Research wishes to improve the reproducibility of the work that we publish. This form provides structure for consistency and transparency in reporting. For further information on Nature Research policies, see Authors & Referees and the Editorial Policy Checklist .

Statistics

For all statistical analyses, confirm that the following items are present in the figure legend, table legend, main text, or Methods section.

n/a	Confirmed	
☐	☒	The exact sample size (*n*) for each experimental group/condition, given as a discrete number and unit of measurement
☐	☒	A statement on whether measurements were taken from distinct samples or whether the same sample was measured repeatedly
☐	☒	The statistical test(s) used AND whether they are one- or two-sided<br>*Only common tests should be described solely by name; describe more complex techniques in the Methods section.*
☐	☒	A description of all covariates tested
☐	☒	A description of any assumptions or corrections, such as tests of normality and adjustment for multiple comparisons
☐	☒	A full description of the statistical parameters including central tendency (e.g. means) or other basic estimates (e.g. regression coefficient) AND variation (e.g. standard deviation) or associated estimates of uncertainty (e.g. confidence intervals)
☐	☒	For null hypothesis testing, the test statistic (e.g. *F*, *t*, *r*) with confidence intervals, effect sizes, degrees of freedom and *P* value noted<br>*Give P values as exact values whenever suitable.*
☒	☐	For Bayesian analysis, information on the choice of priors and Markov chain Monte Carlo settings
☒	☐	For hierarchical and complex designs, identification of the appropriate level for tests and full reporting of outcomes
☒	☐	Estimates of effect sizes (e.g. Cohen's *d*, Pearson's *r*), indicating how they were calculated

Our web collection on statistics for biologists contains articles on many of the points above.

Software and code

Policy information about availability of computer code

Data collection	Diseases associated with IP3R1 as well as functional and network analyses were analyzed at https://www.qiagenbioinformatics.com/products/ingenuitypathway-analysis (Qiagen). For gene ontology (GO) analyses, the list of proteins identified from mass spectrometry was imported to the generic GO term finder at http://go.princeton.edu/ and GO terms were searched against the mouse database. The relevance of IP3R1 to human atherosclerosis was established by searching SNPs in the UK Biobank to identify variants from European and Asia patients, which indicate an association between coronary heart disease or myocardial infarction using the GWAS database. Data on coronary artery disease / myocardial infarction have been contributed by the CARDIoGRAMplusC4D (http://www.cardiogramplusc4d.org/) and UK Biobank Cardio Metabolic Consortium CHD working group using the UK Biobank Resource (application number 9922). Summary level findings from genetic association studies using the GWAS database (https://www.gwascentral.org/) were plotted using LocusZoom (http://locuszoom.org/).
Data analysis	The Mascot version 2.2.6 search engine (Matrix Science) was used to identify proteins from the mass and tandem mass spectra of peptides. Peptide data was matched by searching the UniProtKB human database using the MASCOT engine. Scaffold version 3.6.1 (Proteome Software Inc.) was used to validate MS/MS based peptide and protein identifications. Lesion plaques for aortic roots and arches were quantified using Image J available from NIH website (https://imagej.nih.gov/ij/). Statistical analysis was conducted using GraphPad Prism software (https://www.graphpad.com/scientific-software/prism/).

For manuscripts utilizing custom algorithms or software that are central to the research but not yet described in published literature, software must be made available to editors/reviewers. We strongly encourage code deposition in a community repository (e.g. GitHub). See the Nature Research guidelines for submitting code & software for further information.

Data

Policy information about availability of data

All manuscripts must include a data availability statement. This statement should provide the following information, where applicable:

- Accession codes, unique identifiers, or web links for publicly available datasets
- A list of figures that have associated raw data
- A description of any restrictions on data availability

All data are available from the corresponding author upon reasonable request. Raw data and analysis for mass spectrometry, GO analyses, and atherosclerosis patient information are provided in the supplemental figures tables.

Field-specific reporting

Please select the one below that is the best fit for your research. If you are not sure, read the appropriate sections before making your selection.

[x] Life sciences [ ] Behavioural & social sciences [ ] Ecological, evolutionary & environmental sciences

For a reference copy of the document with all sections, see nature.com/documents/nr-reporting-summary-flat.pdf

Life sciences study design

All studies must disclose on these points even when the disclosure is negative.

Sample size	We used standard sample sizes as reported in the literature for mouse studies. In each group, 5 mice are included and experiments were repeated 3 times. The number of the independent experiments for cell and biological experiments is indicated in the figure legends.
Data exclusions	Sick or diseased animals were excluded.
Replication	For western blotting, we repeated independently conducted experiments at least 3 times.
Randomization	Animals were randomized into groups with the same genotypes, gender, and age.
Blinding	Individuals collecting data or adjudicating outcomes were blinded.

Reporting for specific materials, systems and methods

We require information from authors about some types of materials, experimental systems and methods used in many studies. Here, indicate whether each material, system or method listed is relevant to your study. If you are not sure if a list item applies to your research, read the appropriate section before selecting a response.

Materials & experimental systems

n/a	Involved in the study	
[ ]	[x]	Antibodies
[ ]	[x]	Eukaryotic cell lines
[x]	[ ]	Palaeontology
[ ]	[x]	Animals and other organisms
[ ]	[x]	Human research participants
[x]	[ ]	Clinical data

Methods

n/a	Involved in the study	
[x]	[ ]	ChIP-seq
[ ]	[x]	Flow cytometry
[x]	[ ]	MRI-based neuroimaging

Antibodies

Antibodies used	The antibodies used in this study are listed in a Supplemental Table.
Validation	All antibodies are from commercial sources and were validated by staining with corresponding knockout or knockdown samples.

Eukaryotic cell lines

Policy information about cell lines

Cell line source(s)	HEK 293T cells and HAECs were bought from ATCC. Primary mouse aortic endothelial cells (MAECs) and mouse brain endothelial cells (MBECs) were isolated from aortas or brains according to previous reports.

Authentication	Cells were authenticated by immunostaining, western blot analysis, and FACS.
Mycoplasma contamination	All cell lines in this study tested negative for mycoplasma before their use.
Commonly misidentified lines (See ICLAC register)	No commonly misidentified cell lines were used in this study.

Animals and other organisms

Policy information about studies involving animals; ARRIVE guidelines recommended for reporting animal research

Laboratory animals	All animal protocols were approved by the Institutional Animal Care and Use Committee of Boston Children's Hospital and the Oklahoma Medical Research Foundation (OMRF). ApoE-null mice were purchased from the Jackson Research Laboratory. Epsin and IP3R1 mutant mice were created in our laboratory.
Wild animals	N/A
Field-collected samples	N/A
Ethics oversight	Institutional Animal Care and Use Committee of Boston Children's Hospital and Oklahoma Medical Research Foundation (OMRF).

Note that full information on the approval of the study protocol must also be provided in the manuscript.

Human research participants

Policy information about studies involving human research participants

Population characteristics	Human atherosclerosis patient samples (slides) were obtained from the Maine Medical Center BioBank (MMC BB). De-identified human samples (Supplemental Table 3) obtained from the MMC BB received a 'waiver of consent' from the Oklahoma Medical Research Foundation and Boston Children's Hospital. There was no record linking the subject and the research. Consequently, there is no risk of a breach of confidentiality. Patient samples were initially collected by the MMC BB, which operates under an Institutional Review Board (IRB) approved protocol and is overseen by the MMCRI Office of Research Compliance.
Recruitment	N/A. Only de-identified human tissue slides from the MMC BB were used in this study.
Ethics oversight	Research on human samples obtained from the Maine Medical Center BioBank (MMC BB) was conducted under waiver of consent agreement between Boston Children's Hospital and the Oklahoma Medical Research Foundation. The MMC BB IRB ensures that informed consent was sought from each prospective subject or the subject's legally authorized representative (LAR), in accordance with, and to the extent required by 45 CFR 46.116 and 21 CFR 50.20. In addition, the IRB ensures that informed consent was appropriately documented, in accordance with, and to the extent required by 45 CFR 46.117 and 21 CFR 50.27. The IRB ensured, as part of its review, that the information in the consent document and process was consistent with the research plan, and, when applicable, the HIPAA authorization.

Note that full information on the approval of the study protocol must also be provided in the manuscript.

Flow Cytometry

Plots

Confirm that:

- [x] The axis labels state the marker and fluorochrome used (e.g. CD4-FITC).
- [x] The axis scales are clearly visible. Include numbers along axes only for bottom left plot of group (a 'group' is an analysis of identical markers).
- [x] All plots are contour plots with outliers or pseudocolor plots.
- [x] A numerical value for number of cells or percentage (with statistics) is provided.

Methodology

Sample preparation	MAECs were isolated according methods in Methods and Supplemental Fig. 2. FACS analysis is followed on our standard protocol. In brief, cells were staining with anti-VE-Cadhesin antibody in PBS buffer containing 1% FBS for 45 min to 1 hour at 4°C in dark. After the staining, the cells were washed and resuspended in 400uL PBS, and then acquired with FACS for data collection.
Instrument	Guava easyCyte 6HT/2L flow cytometer, Millipore Corporation, Billerica, MA
Software	FlowJo software (Tree Star Inc., Ashland, OR).

Cell population abundance	Purity is over 90%, compared to negative control
Gating strategy	Using forward scatter (FSC) and side scatter (SSC), we started with a negative selection strategy. As a general guide, this can often be done by size, which is estimated by forward scatter – cellular debris is usually FSC-low and dead cells often have lower forward scatter and higher side scatter than live cells. By drawing a gate that excludes those events with low FSC and high SSC, we can exclude debris and dead cells from the analysis.

☒ Tick this box to confirm that a figure exemplifying the gating strategy is provided in the Supplementary Information.
