# Supplementary material for: Epsin-mediated degradation of IP3R1 fuels atherosclerosis
Source: Nat Commun. 2020 Aug 7;11:3984. doi: 10.1038/s41467-020-17848-4 (PMC7414107; doi:10.1038/s41467-020-17848-4)
Supplement: Supplementary file 7 — Description of Additional Supplementary Files [file 41467_2020_17848_MOESM7_ESM.pdf]

Title: Supplementary Data file 1
Description: Liquid Chromatography with tandem mass spectrometry (LC/MS-MS) proteomic data. Proteins identified in the epsin 1 immunocomplex and IgG control data are shown from top to bottom. The IgG control gel is shown below the IgG control data.

Title: Supplementary Data file 2
Description: Gene ontology (GO) analysis data. The GO aspects of biological process, cellular component, molecular function, and a summary are shown from top to bottom.

Title: Supplementary Data file 3
Description: Medical information for human atherosclerosis patient samples. Specific diagnostic information is listed below the summary chart.

Title: Supplementary Data file 4
Description: List of key resources. Sources of antibodies, reagents, experimental models, recombinant vectors, and PCR primers are shown.
