# Supplementary material for: Epsin-mediated degradation of IP3R1 fuels atherosclerosis
Source: Nat Commun. 2020 Aug 7;11:3984. doi: 10.1038/s41467-020-17848-4 (PMC7414107; doi:10.1038/s41467-020-17848-4)
Supplement: Supplementary file 9 — Source Data [file 41467_2020_17848_MOESM9_ESM.zip › Dong NCOMM-19-1522299B Uncropped Western Blots.pdf]

Uncropped gel blot

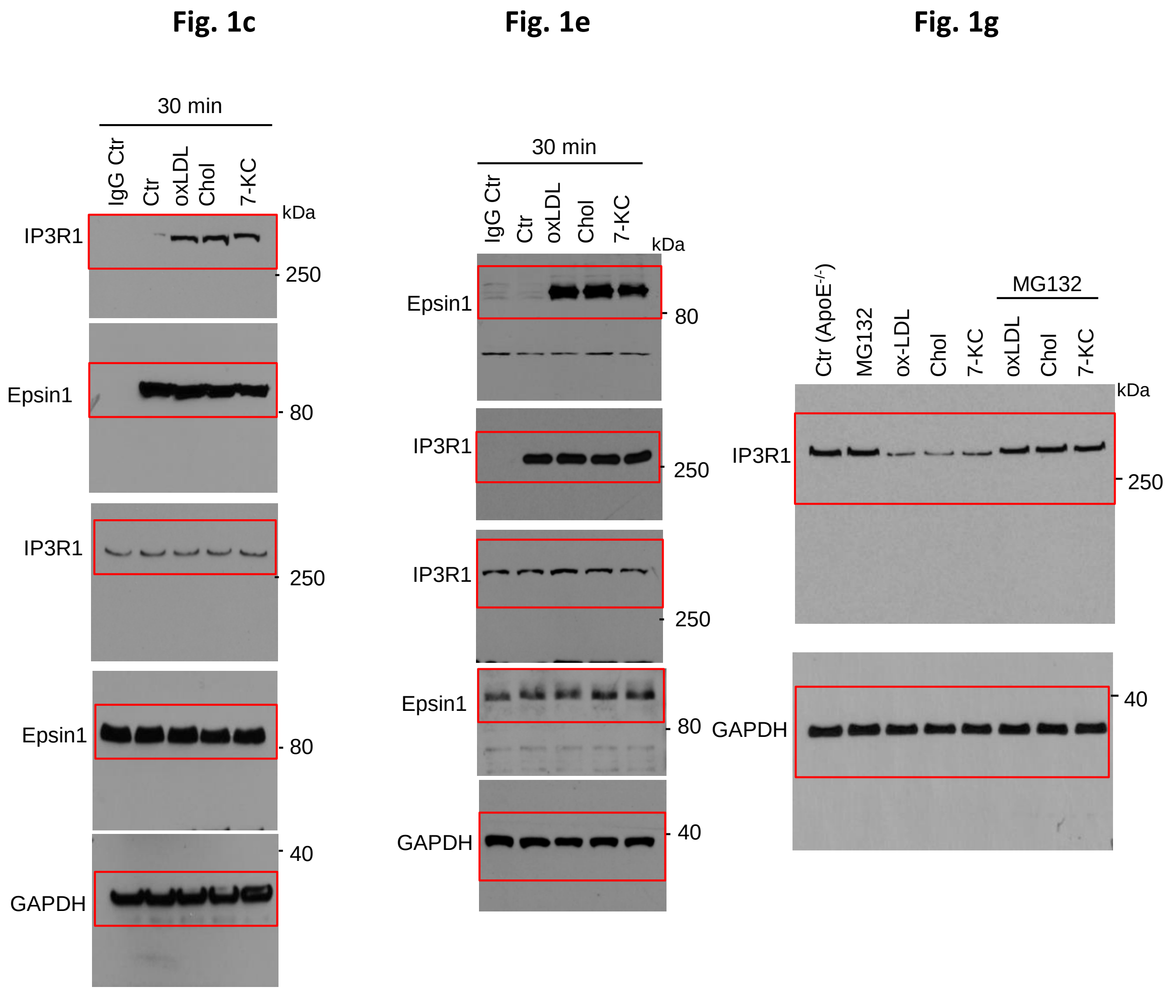

Figure-1 (c, e, g)

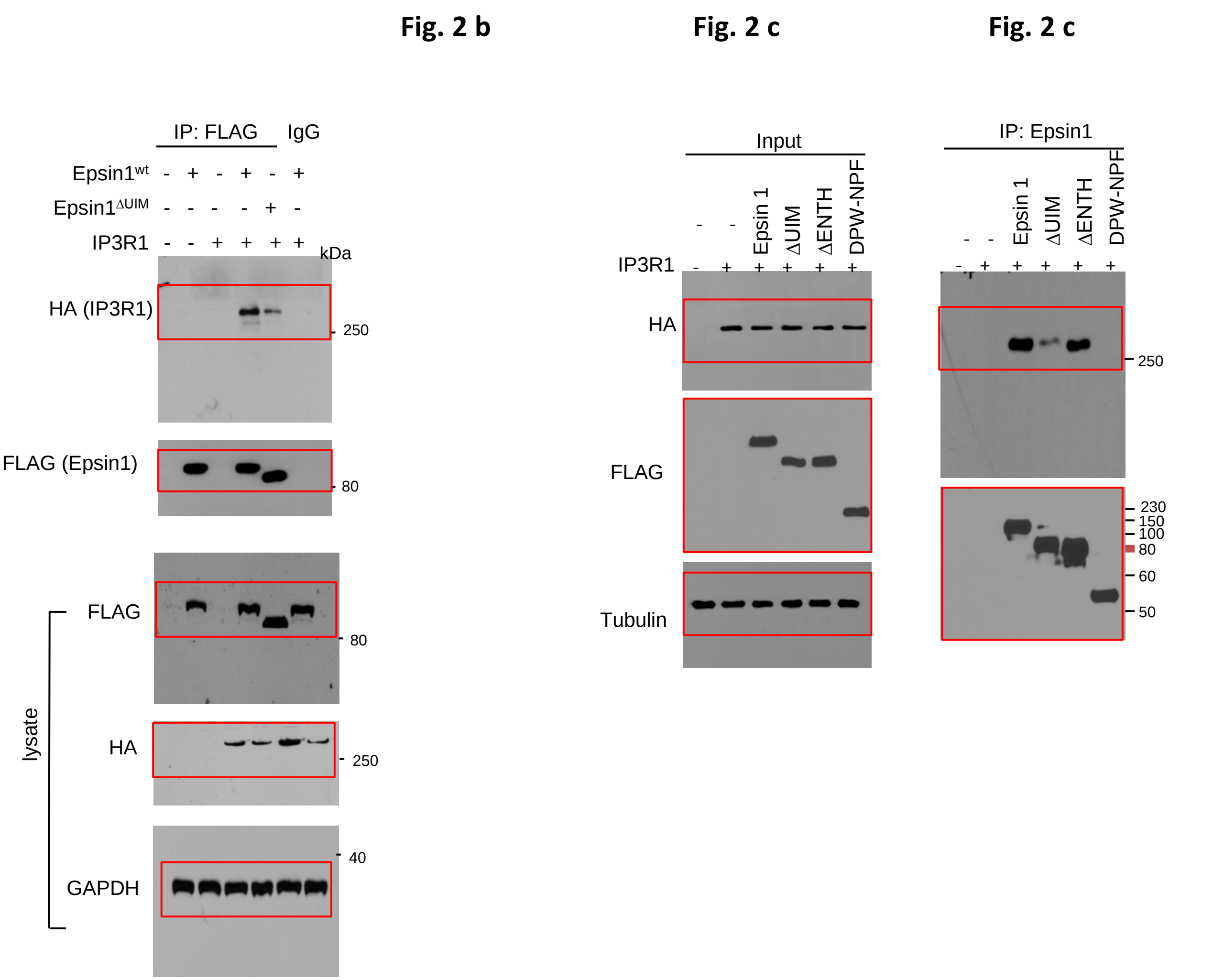

Fig. 2 b
Fig. 2 c
Fig. 2 c
IP: FLAG
IgG
Epsin1wt
Epsin1ΔUIM
IP3R1
kDa
HA (IP3R1)
250
FLAG (Epsin1)
80
FLAG
80
lysate
HA
250
40
GAPDH
Input
Epsin 1
ΔUIM
ΔENTH
DPW-NPF
IP3R1
HA
FLAG
Tubulin
IP: Epsin1
Epsin 1
ΔUIM
ΔENTH
DPW-NPF
250
230
150
100
80
60
50

Figure-2 (b, c)

Fig. 2 e

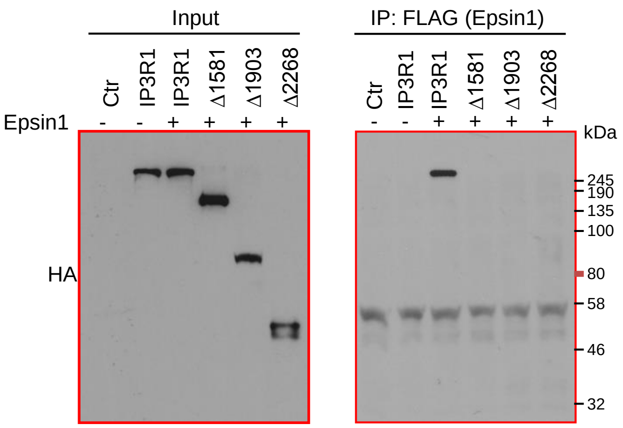

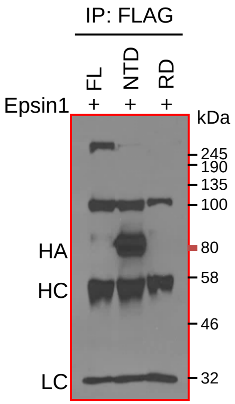

Fig. 2 f

Fig. 2 h

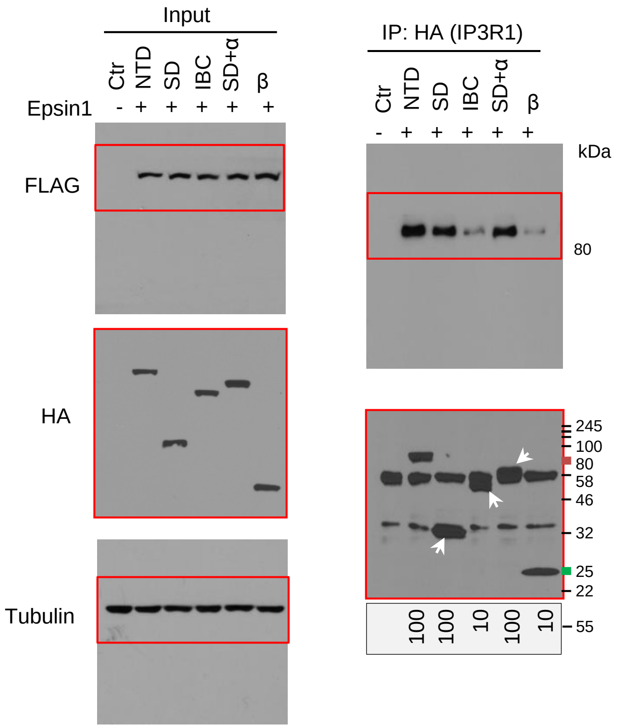

Figure-2 (e, f, h)

Fig. 3a

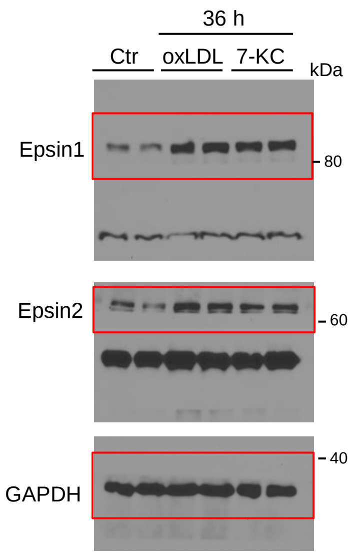

Fig. 3c

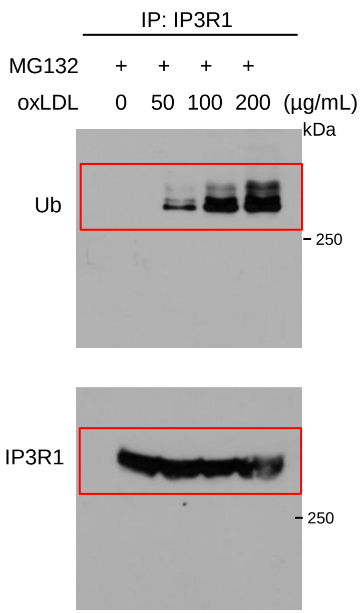

Fig. 3d

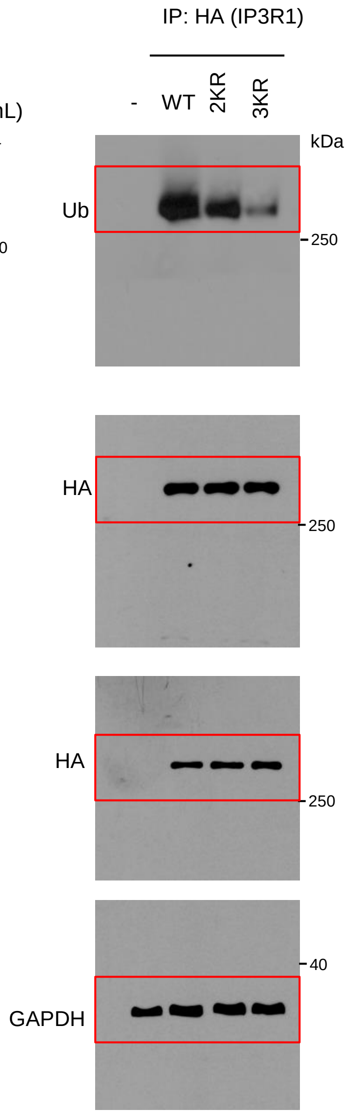

Fig. 3e **Fig. 3f** **Fig. 3g**

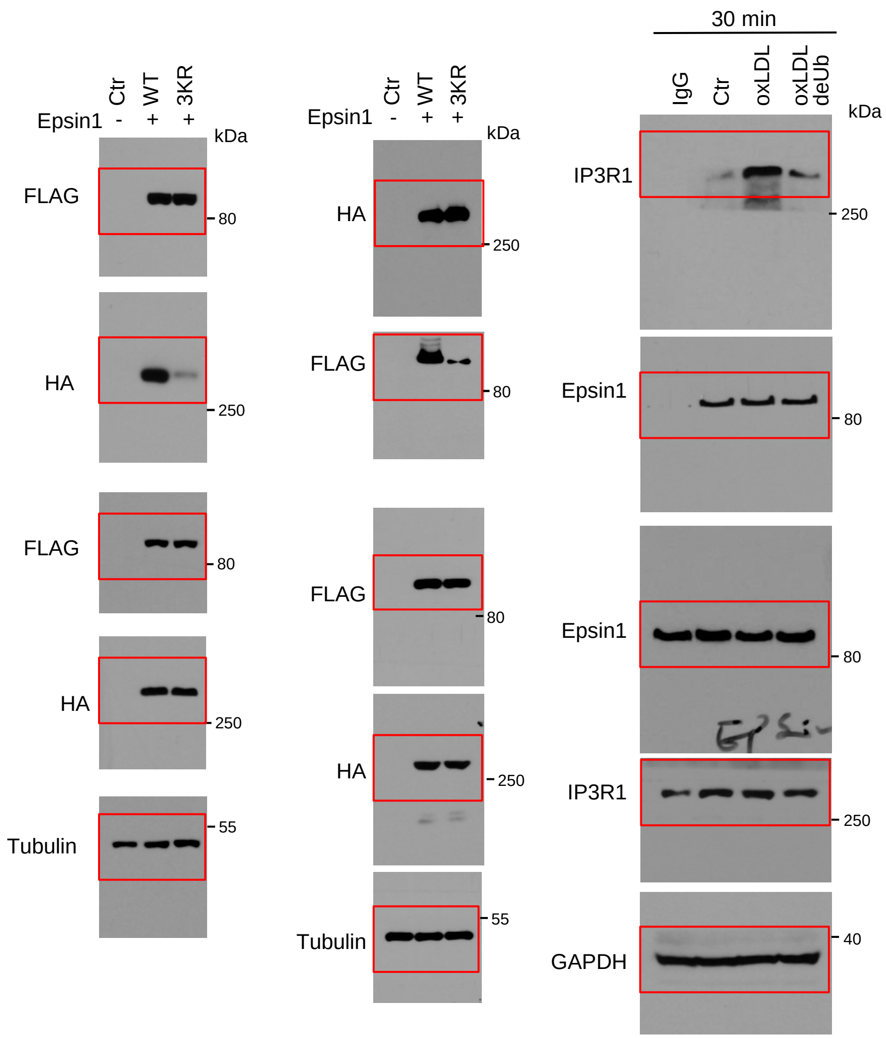

Figure-3 (e, f, g)

Fig. 3h

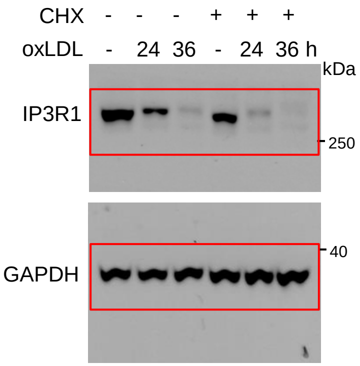

Fig. 3j

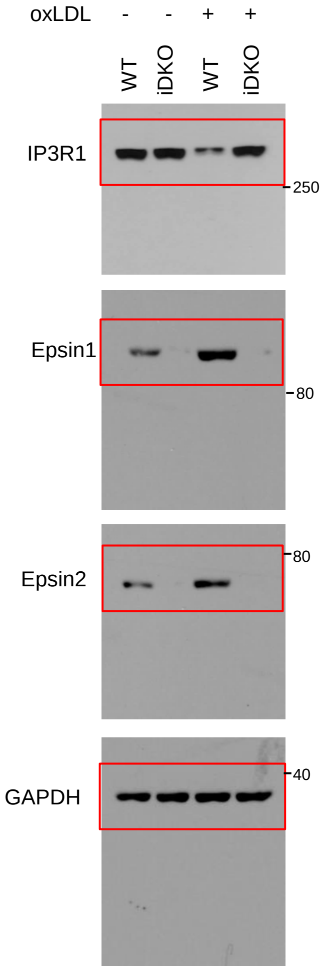

Fig. 3l

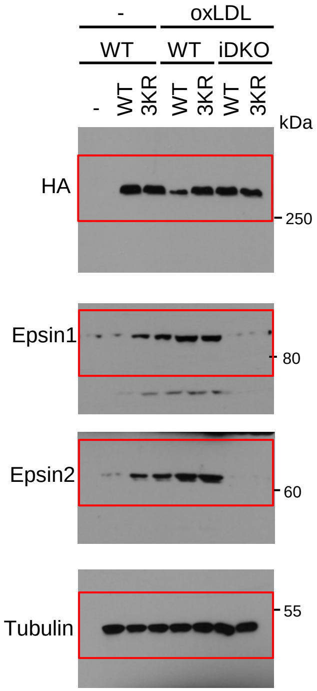

Figure-3 (h, j, l)

Fig. 3m

Fig. 3m

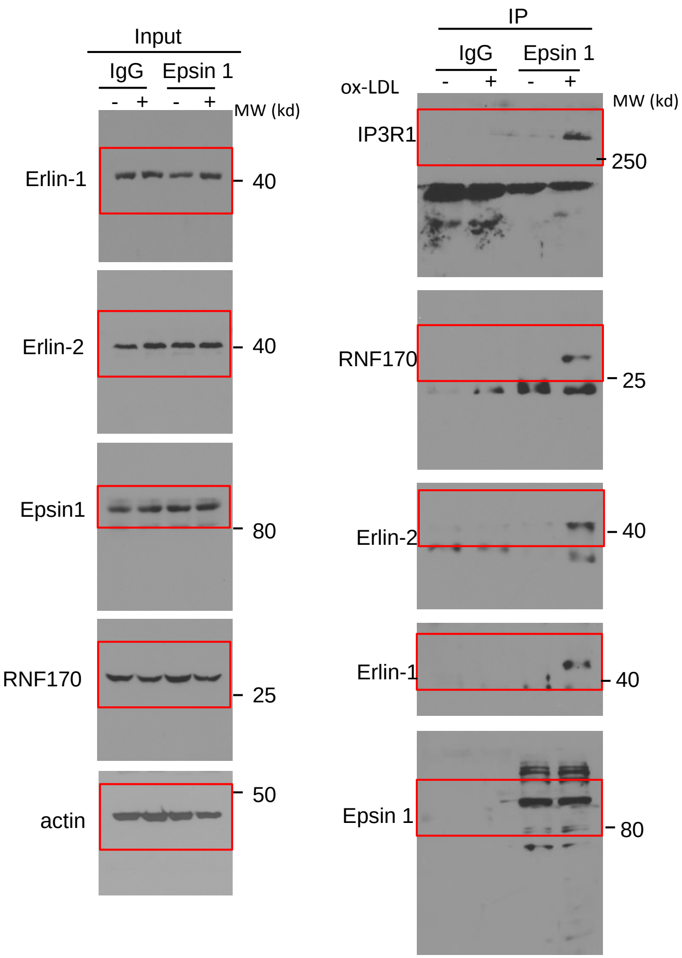

Figure-3 (m)

Fig. 3 n

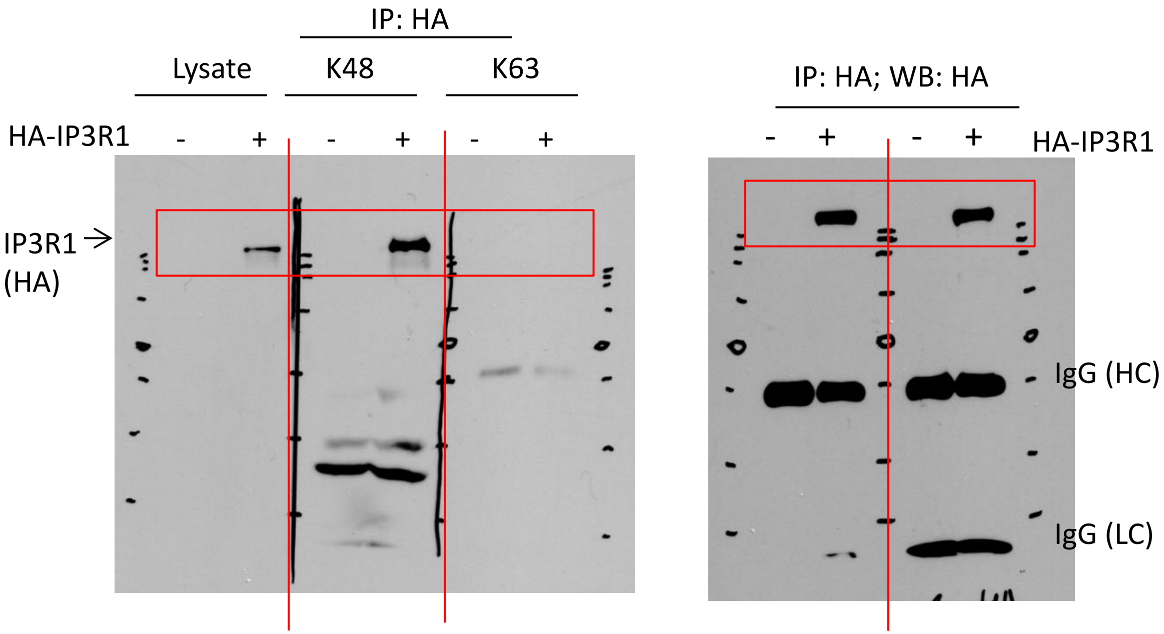

Note: NC membranes were spliced as indicated in the middle of protein markers after SDS-PAGE electrophoresis, followed by the incubation with different primary antibodies HA, anti-K48 and anti-K63 respectively, and then 2nd antibodies. The spliced pieces of NC membranes were then merged together (according to protein markers) for X-film exposure.

Figure-3 (n)

Fig. 4d

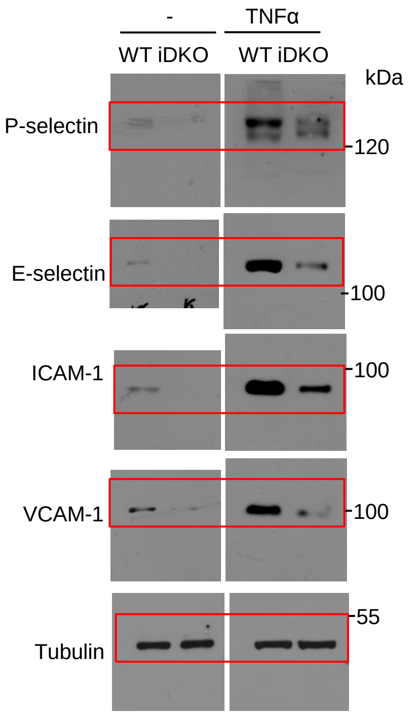

Figure-4 (d)

Fig. 5f

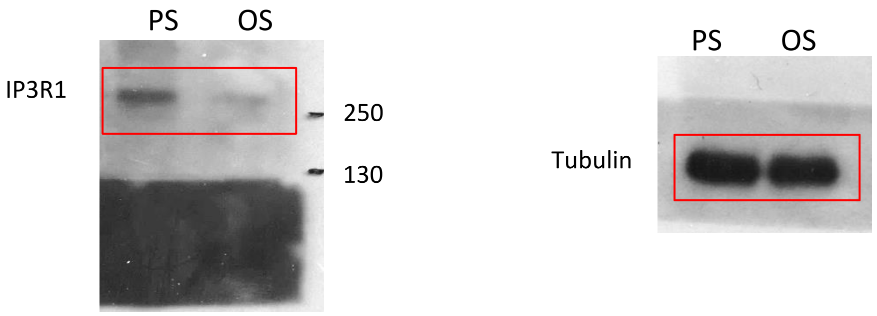

Fig. 5h

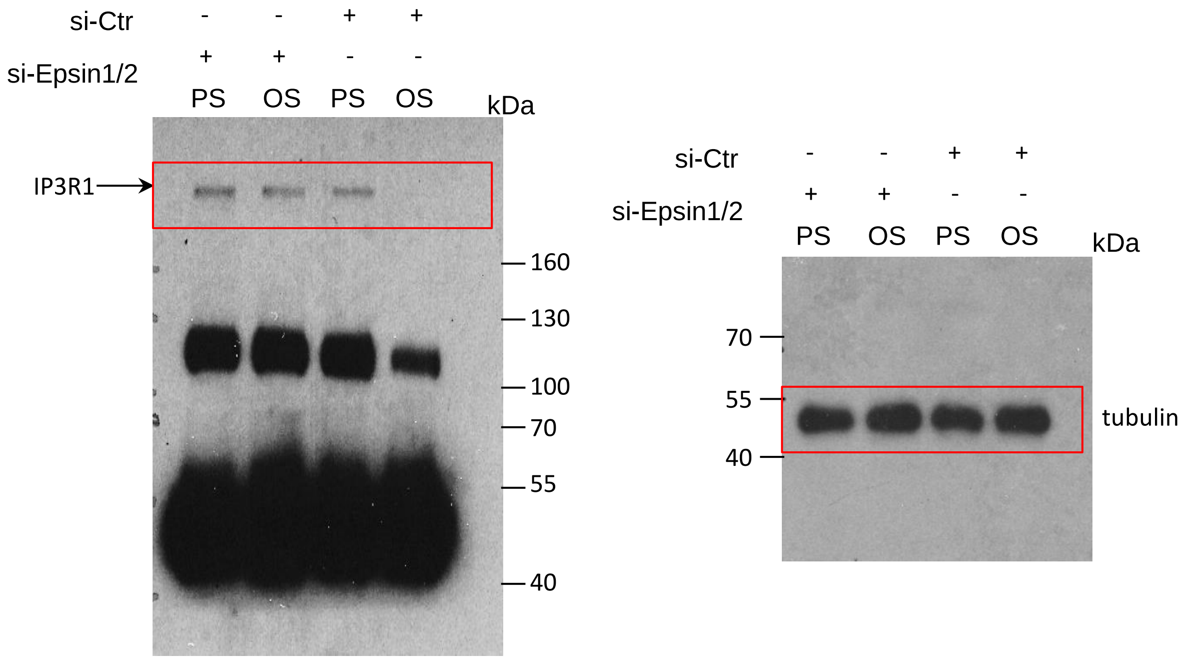

Figure-7 (f, h)

Supple Fig 2c

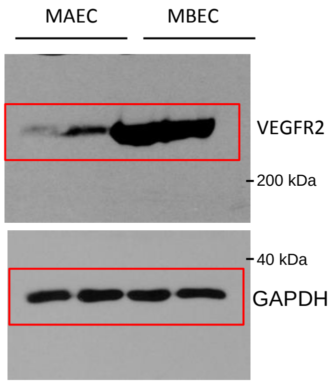

Supple Fig 2h

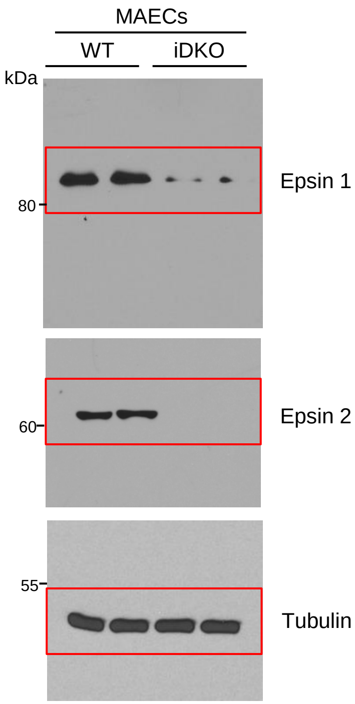

Supplement Figure- 2 (c, h)

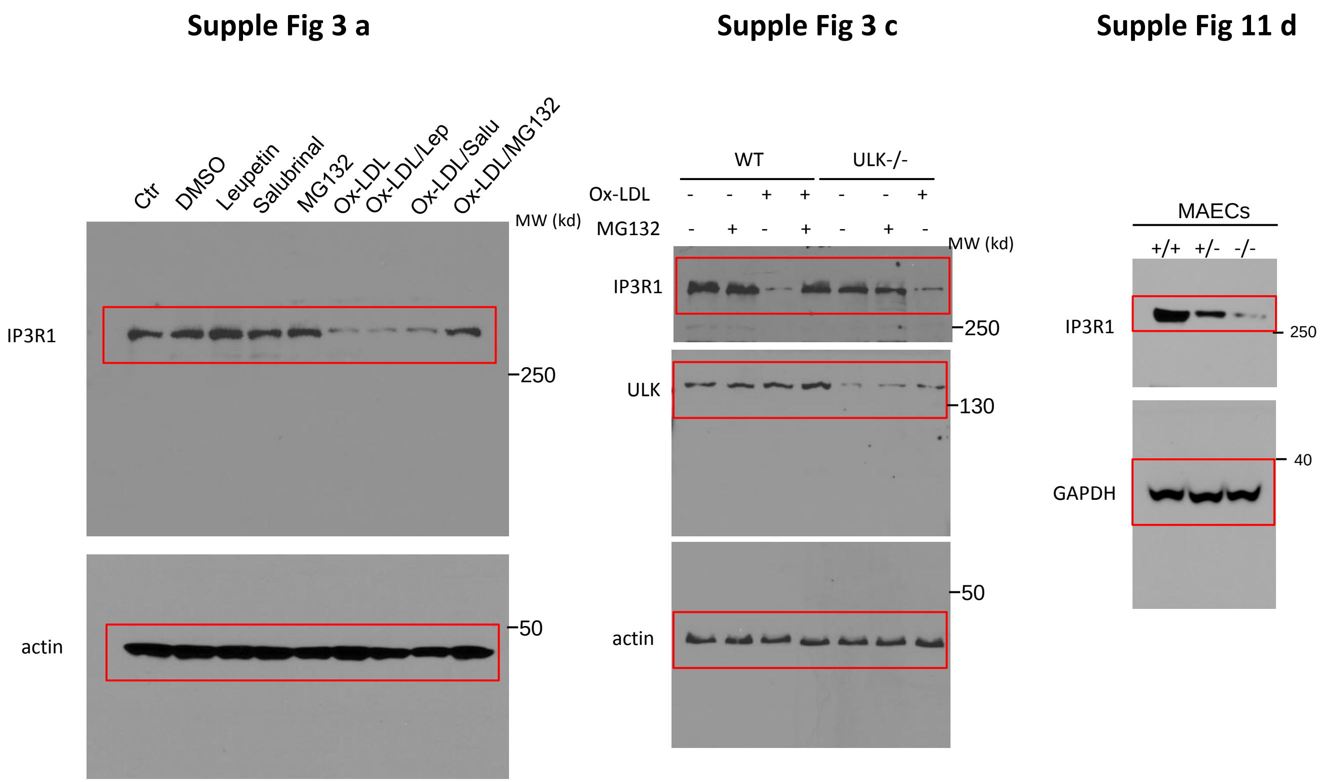

Supplement Figure-3 (a, c) and Figure 11d
